# Fibrosis status, extrahepatic multimorbidity and all-cause mortality in 53,093 women and 74,377 men with metabolic dysfunction associated steatotic liver disease (MASLD) in UK biobank

**DOI:** 10.1186/s12876-025-04079-4

**Published:** 2025-07-31

**Authors:** Qi Feng, Chioma N. Izzi-Engbeaya, Pinelopi Manousou, Mark Woodward

**Affiliations:** 1 The George Institute for Global Health (UK), School of Public Health, Faculty of Medicine, Imperial College London, Scale Space, 58 Wood Lane, London, W12 7RZ UK; 2 Section of Investigative Medicine and Endocrinology, Department of Metabolism, Digestion and Reproduction, Faculty of Medicine, Imperial College London, London, UK; 3 Division of Digestive Diseases, Department of Metabolism, Digestion and Reproduction, Faculty of Medicine, Imperial College London, London, UK; 4 Department of Hepatology, St Mary’s Hospital, Imperial College Healthcare NHS Trust, London, UK; 5 The George Institute for Global Health (Australia), University of South New Wales, Sydney, Australia

**Keywords:** MASLD, Fibrosis, FIB4, Multimorbidity, Mortality, UK biobank

## Abstract

**Introduction:**

People with metabolic-dysfunction associated steatotic liver disease (MASLD) had higher risk of extrahepatic multimorbidity, and fibrosis is the strongest prognostic factor for mortality in MASLD. This study aimed to investigate how fibrosis was associated with multimorbidity and their relationships with all-cause mortality.

**Methods:**

We utilized data from the UK Biobank. MASLD was identified via a Fatty Liver Index ≥ 60 and the presence of cardiometabolic risk factors. The fibrosis-4 (FIB4) score was used to measure liver fibrosis. Multimorbidity was defined as having two or more long-term conditions (LTCs) from a prespecified list of 47 LTCs. Logistic regressions estimated the cross-sectional association between FIB4 scores and multimorbidity prevalence, while Cox models assessed the prospective association between FIB4 scores, multimorbidity and mortality.

**Results:**

We included 127,470 participants with MASLD (41.7% female, age 57.4 years, 21.3% with multimorbidity, 2.2% with high FIB4 scores). 14,471 deaths were recorded during 13-year follow-up. Compared to low FIB4 scores, high FIB4 scores were associated with 41% higher prevalence of multimorbidity (OR 1.41 (95%CI 1.30–1.54)), and 94% higher all-cause mortality (HR 1.94 (95%CI 1.77–2.13)), while adjusting for multimorbidity reduced the association by 10%, primarily driven by contributions from cardiovascular diseases, extrahepatic cancers, and chronic kidney disease.

**Conclusion:**

FIB4 scores were positively associated with higher multimorbidity and mortality in MASLD patients. Adjustment for multimorbidity reduced 10% the relationship between fibrosis and mortality, with cardiovascular diseases, cancers and chronic kidney disease contributing notably to this reduction. These findings underscore the importance of managing both fibrosis and multimorbidity in MASLD patients.

**Supplementary Information:**

The online version contains supplementary material available at 10.1186/s12876-025-04079-4.

## Introduction

Metabolic-dysfunction associated steatotic liver disease (MASLD), formerly known as non-alcoholic fatty liver disease (NAFLD), is the most common chronic liver disease, with an estimated global prevalence of 32.4% [1]. MASLD is linked to a range of hepatic and extra-hepatic diseases, particularly cardiovascular diseases (CVDs) and extrahepatic cancers, and chronic kidney disease [2, 3], resulting in a higher risk of extrahepatic multimorbidity (defined as having two or more long-term conditions (LTCs)) [4].

Fibrosis status is the most important prognostic factor for severe liver diseases and mortality in people with MASLD [5]. A systematic review has shown that biopsy-confirmed advanced fibrosis stage increased mortality by 2.5 times, compared to early stage fibrosis [6]. Assessing liver fibrosis stages is therefore pivotal in MASLD management, with liver biopsy being the gold standard. However, liver biopsy is invasive, costly, requires significant clinical skills, carries extra risk of complications, and is prone to sampling variability, thus making it impractical for large cohort studies. Consequently, various non-invasive assessments have been developed and adopted in international guidelines for MASLD management [7, 8].

Currently, there has been a lack of evidence regarding the association between liver fibrosis and multimorbidity, and how their interplay is associated with all-cause mortality. Therefore, this study was aimed to investigate whether liver fibrosis status was associated with multimorbidity, and the potential role of multimorbidity in the association between fibrosis and mortality in people with MASLD.

## Methods

### Data and participants

UK Biobank is a prospective cohort of half million participants aged between 40 and 70 years old recruited across the UK between 2006 and 2010. During baseline assessment, data were collected on socioeconomic characteristics, lifestyle, health status, anthropometry and physical measures. The participants were followed up via linkage to national registries, and hospitalisation datasets. All participants provided informed consent.

We identified participants with high risk of MASLD using the definition proposed by Rinella M. et al. [9], considering liver steatosis, cardiometabolic risk factors and low alcohol consumption (< 20/30 g/day for female/male). We used a fatty liver index (FLI) ≥ 60 as a proxy for liver steatosis. FLI is a non-invasive biomarker for hepatic steatosis, calculated from body mass index (BMI), waist circumference, triglycerides, and gamma-glutamyl transferase (GGT) [10]. The cutoff value of 60 to define liver steatosis has been validated and used previously [11, 12]. We considered the following five cardiometabolic risk factors (CMRF): overweight/obesity (BMI ≥ 25 kg/m² AND/OR waist circumference > 94/80 cm (male/female)), prediabetes/diabetes (glycated haemoglobin (HbA1c) ≥ 39 mmol/mol AND/OR diagnosis of type 2 diabetes AND/OR on use of insulin), hypertension (systolic blood pressure (BP) ≥ 130 AND/OR diastolic BP ≥ 85 mmHg AND/OR on antihypertensive drug treatment or diagnosis of hypertension), high triglycerides (TG) (plasma TG ≥ 1.70 mmol/L AND/OR on use of statin), and low high-density lipoprotein (HDL) cholesterol (HDL-cholesterol ≤ 1.0/1.3 mmol/L (male/female)) AND/OR on use of statin).

We excluded women who were pregnant at baseline, and people who had missing data for calculating FLI or defining MASLD. We further removed those with alcohol related liver disease (ALD), metabolic-dysfunction and alcohol related liver disease (MetALD), or other chronic liver disease (including viral hepatitis, liver fibrosis, liver cirrhosis, hepatocellular carcinoma, hemochromatosis, Wilson’s disease, biliary cirrhosis, autoimmune hepatitis, primary sclerosing cholangitis, drug-induced liver injury and Budd-Chiari syndrome). For ALD and MetALD, we used the definitions proposed by Rinella et al. [9], while other chronic liver disease was ascertained via self-report and hospitalisation data; the code lists for these conditions are presented in supplementary methods. We excluded individuals with clinically diagnosed fibrosis or cirrhosis at baseline, because such diagnoses may reflect underlying chronic liver conditions other than steatosis, such as viral hepatitis or autoimmune liver disease.

We used the FIB4 score, a non-invasive surrogate marker for fibrosis status and linked to increased mortality [13, 14], which required age, serum aspartate aminotransferase and alanine aminotransferase levels, and platelet count [15]. We used the cutoff values 1.30 and 2.67 to categorise low, intermediate and high levels of FIB4 score for people < 65 years old, and 2.00 and 2.67 for people ≥ 65 years old [16]. Previous research in Western populations has shown that, for individuals under 65 years, FIB4 cutoff values of 1.30 and 2.67 yielded sensitivities of 90% and 30%, and specificities of 61% and 97.6%, respectively. For individuals over 65 years, cutoff values of 2.0 and 2.67 yielded sensitivities of 77% and 53%, and specificities of 70% and 85%, respectively [17]. Among people with MASLD, we further excluded those who had missing FIB4 data.

### Variable measurement

Multimorbidity is defined as having two or more LTCs from a prespecified list of 47 LTCs (Table 1), in line with widely used definitions in population health research and recommendations by the UK Academy of Medical Sciences [18]. We used a modified list of LTCs identified in a three-round Delphi study of 25 public participants and 150 healthcare professionals (including clinicians, researchers and policy makers) in 2021 [19]. The criteria for including these LTCs included their impacts on risk of death, quality of life, frailty, physical disability, mental health and treatment burden. Since MASLD is our index disease, we define multimorbidity excluding MASLD and its associated CMRFs. Among the conditions identified in this Delphi study, we further removed two liver conditions (hepatocellular carcinoma and chronic liver disease), as well as post-acute covid-19 (because this was only included in ICD10 after October 2021, long after the initiation of UK Biobank) and chronic Lyme disease (because it is rare in UK population). The conditions covered extrahepatic solid organ cancers, haematological cancers, cardiovascular, metabolic/endocrine, respiratory, digestive, renal, mental/behavioural and congenital conditions. We used the self-reported data and hospitalisation records for phenotyping multimorbidity (up to February 2018 for participants from Wales and March 2021 for participants from England or Scotland). The codelists are shown in the supplementary method. We also assessed prevalence of multimorbidity at baseline and annually up to 13 years after baseline (the median follow-up time).

**Table 1:** Long-term conditions included for multimorbidity phenotyping

Categories	Conditions
Congenital & genetic (3)	Congenital diseases, chromosomal abnormalities, cystic fibrosis
Infections (3)	HIV/AIDS, chronic urinary tract infection, tuberculosis
Mental/behavioural (11)	Parkinson disease, dementia, epilepsy, schizophrenia, depression, anxiety, bipolar disorder, substance use disorder, eating disorder, autism, post-traumatic stress disorder (PTSD)
Cardiovascular (8)	Ischemic heart disease, heart failure, stroke, transient ischemic attack (TIA), heart valve disorder, arrythmia, venous thromboembolism, aneurysm
Respiratory (3)	Chronic obstructive pulmonary disease (COPD), asthma, other chronic respiratory disease
Cancer (2)	Extrahepatic solid organ cancers, haematological cancers
Metabolic/endocrine (3)	Addison’s disease, thyroid disorder, gout
Digestive (3)	Chronic pancreatitis, peptic ulcer disease, irritable bowel disease
Renal (1)	Chronic kidney disease
Musculoskeletal (4)	Osteoarthritis, osteoporosis, paralysis, connective tissue diseases
Sensory (3)	Vision impairment, hearing impairment, Meniere’s disease
Others (3)	Anaemia, multiple sclerosis, endometriosis

The primary outcome of interest was all-cause mortality, confirmed via national death registry records. Participants were censored at the date of death or the last date of follow-up (30 November 2022), whichever occurred first. We also assessed incidence of multimorbidity in individuals with 0–1 LTC at baseline.

Ethnicity was classified into White, Asian, Black, and mixed/others. The Townsend Deprivation Index is a postcode-derived measure used to designate socioeconomic status. Lifestyle factors considered were smoking status (current, previous and never smoker), alcohol consumption, and physical activity. Alcohol consumption was assessed via weekly or monthly consumptions of red wine, white wine, champagne, beer, spirits, fortified wine and other alcoholic drinks; the consumption was converted to standard UK alcohol units and grams and summed up to derive the average daily alcohol consumption (g/day) [20]. Physical activity level was categorized into low, moderate and high levels, based on the frequency, duration and intensity of their physical activities. Systolic and diastolic blood pressure were measured by trained staff. Blood biochemistry markers included TG, HDL cholesterol, HbA1c and platelet. For all the categorical covariates, answers of “unknown”, “do not know”, “prefer not to say” were combined into one “unknown” category.

### Statistical analysis

The baseline characteristics of participants were summarised using mean with standard deviation (SD) or median with interquartile range (IQR), as appropriate, and frequency with percentage, stratified by FIB4 score levels.

We used bar charts to demonstrate the distribution of the number of LTCs stratified by FIB4 score levels. We used line plot to visualise the trajectory of multimorbidity prevalence during follow-up. We calculated the prevalence (per 1000) for each LTC at baseline and ranked them in people with low, intermediate and high FIB4 scores, for males and females separately. We fitted logistic regression, adjusted for sex, age, ethnicity, education and Townsend Deprivation Index (in fifths), to estimate prevalence odds ratios (ORs) and their 95% confidence intervals (CIs) relating cross-sectionally FIB4 score levels and prevalence of multimorbidity (defined as having ≥ 2 LTCs) at baseline. Similar logistic regression models were also fitted to estimate association between FIB4 score levels and each LTC; p values were corrected for multiple testing using false discovery rate (FDR) method [21], with Benjamini-Hochberg procedure, implemented using the p.adjust() function in R software. Associations with an FDR-adjusted p value < 0.05 were considered statistically significant. We estimated the association between FIB4 score levels and multimorbidity in subgroups stratified by age (< 65, ≥ 65), Townsend Deprivation Index (fifths), education, smoking, physical activity level, BMI levels (< 25, 25–30, ≥ 30) and diabetes. To examine the prospective association between FIB4 scores and incident multimorbidity, we fitted Cox proportional hazard model in people with 0 or 1 LTC at baseline, respectively, adjusted for age, sex, ethnicity, education and Townsend Deprivation Index (in fifths).

Cox proportional hazard regression models were used to assess the prospective association between FIB4 score levels and all-cause mortality, expressed as hazard ratio (HR) and 95% CI, using a low FIB4 score level as reference. Models were stratified by region and age group, and adjusted for sex, ethnicity, education, Townsend Deprivation Index (in fifths), physical activity level, smoking status, and alcohol consumption. The proportional hazard assumption was examined by scaled Schoenfeld residuals, and no evidence was observed for its violation. Relative HR (RHR) between sex (males to females) was estimated via fitting an interaction term (sex*FIBF group) in the adjusted Cox model. To test whether the association of FIB4 score levels was independent of multimorbidity, we additionally adjusted for multimorbidity in the model to see whether it would change the coefficient estimates, and if yes, by how much; we calculated the reduction in coefficients before and after additional adjustment of multimorbidity. Similarly, we also adjusted for each LTC one by one in the Cox model, and to see by how much the association estimate would change after additional adjustment.

We also calculated the multimorbidity-adjusted associations between FIB4 score levels and all-cause mortality in subgroups stratified by age, Townsend Deprivation Index, education, smoking status, physical activity level, BMI levels, multimorbidity status, and diabetes. For sensitivity analysis, we (1) excluded the first two years of follow-up when estimating associations with all-cause mortality; and (2) used NAFLD fibrosis score to assess fibrosis, with cutoff values of -1.455 and 0.676 for low, intermediate and high levels [22], and re-assessed the associations between fibrosis, multimorbidity and mortality.

All analyses were conducted in R.

## Results

### Baseline characteristics

**Fig. 1:**
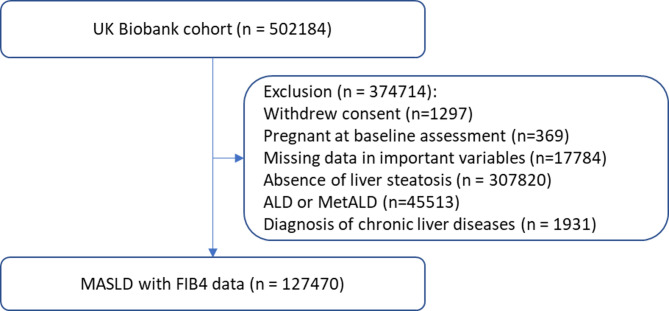
The flowchart of participant selection. MASLD: metabolic dysfunction-associated steatotic liver disease. ALD: alcohol related liver disease. MetALD: metabolic dysfunction and alcohol related liver disease All data as recorded at baseline

We included 127,470 participants (41.7% females, mean age 57.4 years) (Fig. 1). Most of the participants had low FIB4 scores (70.6%), and 2.2% had high FIB4 scores, while one participant had insufficient data to calculate FIB4. Table 2 shows the baseline characteristics. Compared to people with high FIB4 scores, those with lower FIB4 scores were more likely to be younger, female, better educated, physically inactive, and less likely to smoke or consume alcohol. They also had lower waist circumference, lower systolic blood pressure but higher diastolic blood pressure and platelet count. More details on baseline characteristics are shown in supplementary Table 1.

**Table 2:** Baseline characteristics of participants with baseline MASLD by FIB-4 score levels

	FIB4 low	FIB4 intermediate	FIB4 high	Overall
	*n* = 90,035 (70.6%)	*n* = 34,669 (27.2%)	*n* = 2766 (2.2%)	*n* = 127,470 (100.0%)
Sex, female	41,343 (45.9%)	11,001 (31.7%)	748 (27.0%)	53,093 (41.7%)
Age, years	56.6 (8.4)	59.3 (5.6)	62.9 (5.7)	57.4 (7.9)
Townsend deprivation index 1st fifth (least deprived)	17,698 (19.7%)	7323 (21.1%)	541 (19.6%)	25,562 (20.1%)
5th fifth (most deprived)	18,328 (20.4%)	6511 (18.8%)	555 (20.1%)	25,394 (19.9%)
Education, higher education	23,365 (26.0%)	9231 (26.6%)	640 (23.1%)	33,236 (26.1%)
Ethnicity, white	83,828 (93.1%)	32,297 (93.2%)	2579 (93.2%)	118,704 (93.1%)
Smoking, never	48,103 (53.4%)	17,831 (51.4%)	1327 (48.0%)	67,261 (52.8%)
Alcohol drinking, g/day	7.9 (8.8)	9.3 (9.2)	9.2 (9.2)	8.3 (8.9)
Physical activity, high	22,043 (24.5%)	9696 (28.0%)	797 (28.8%)	32,536 (25.5%)
FIB4 score	1.1 (0.3)	1.7 (0.3)	4.7 (20.3)	1.3 (3.1)
ALT, log_10_ U/L	1.4 (0.2)	1.4 (0.2)	1.5 (0.3)	1.4 (0.2)
AST, log_10_ U/L	1.4 (0.1)	1.5 (0.1)	1.6 (0.2)	1.4 (0.1)
GGT, log_10_ U/L	1.6 (0.3)	1.6 (0.3)	1.7 (0.4)	1.6 (0.3)
BMI, kg/m^2^	31.9 (4.7)	31.4 (4.4)	31.5 (4.6)	31.8 (4.6)
Waist circumference, cm	102.4 (10.2)	103.0 (10.1)	104.2 (10.6)	102.6 (10.2)
Waist-to-height ratio, %	60.7 (6.2)	60.2 (6.0)	60.9 (6.4)	60.6 (6.2)
Systolic blood pressure, mmHg	141.2 (17.4)	142.5 (17.6)	143.8 (18.6)	141.6 (17.5)
Diastolic blood pressure, mmHg	85.2 (9.7)	85.0 (9.8)	83.8 (10.3)	85.1 (9.7)
Triglycerides, mmol/L	2.2 [1.3]	2.1 [1.3]	2.0 [1.3]	2.1 [1.3]
HDL cholesterol, mmol/L	1.1 (0.4)	1.1 (0.4)	1.1 (0.4)	1.1 (0.4)
HbA1c, mmol/mol	37.3 (10.1)	36.8 (9.8)	38.2 (11.9)	37.2 (10.1)
Platelet, 10^9^ cells/L	274.7 (56.8)	209.8 (38.7)	149.6 (51.6)	254.4 (61.8)
Hypertension	72,094 (80.1%)	28,023 (80.8%)	2235 (80.8%)	102,352 (80.3%)
Obesity	89,089 (98.9%)	34,234 (98.7%)	2706 (97.8%)	126,029 (98.9%)
Diabetes	28,339 (31.5%)	10,350 (29.9%)	1047 (37.9%)	39,736 (31.2%)
High TG	66,192 (73.5%)	24,639 (71.1%)	1788 (64.6%)	92,619 (72.7%)
Low HDL	43,112 (47.9%)	14,955 (43.1%)	1323 (47.8%)	59,390 (46.6%)

HbA1c: glycated hemoglobin. HDL: high density lipoprotein. Number(number): mean(SD). Number(percent%): frequency(percent). Number[number]: median[interquartile range]. FIB-4 score levels were defined using the cutoff values 1.30 and 2.67 to categorise low, intermediate and high levels for people < 65 years old, and 2.00 and 2.67 for people > = 65 years old

### FIB4 scores and Multimorbidity

**Fig. 2:**
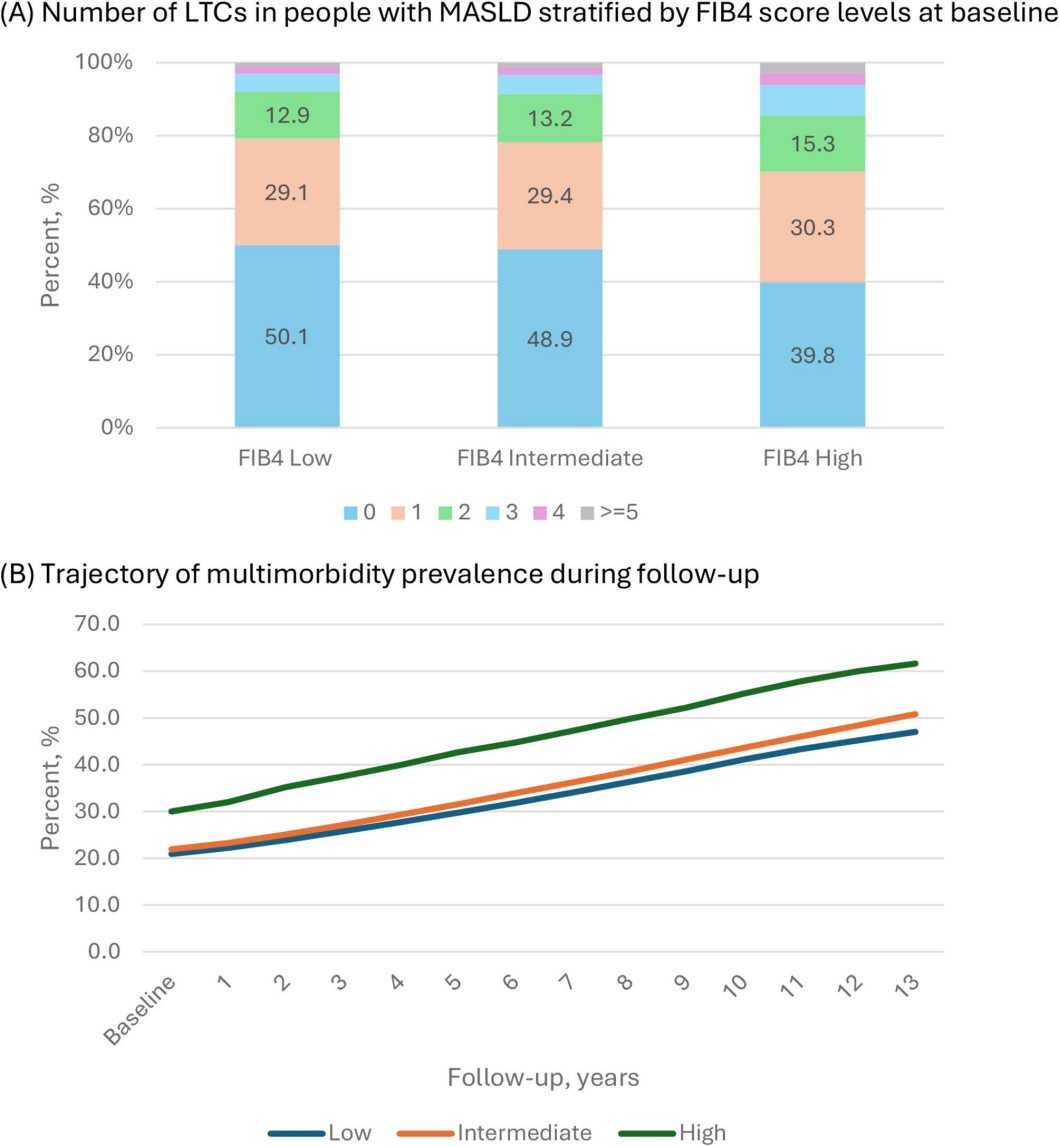
LTC distribution at baseline and trajectory of multimorbidity prevalence during follow-up. FIB-4 score levels were defined using the cutoff values 1.30 and 2.67 to categorise low, intermediate and high levels for people < 65 years old, and 2.00 and 2.67 for people > = 65 years old. LTC: long-term condition

At baseline, 49.5% participants had no prevalent LTC, while 21.3% had prevalent multimorbidity (≥ 2 LTCs). The prevalence of multimorbidity was notably higher among people with high FIB4 scores (29.9%) than those with low (20.8%) or intermediate (21.8%) scores (Fig. 2(A)). Multimorbidity was also more common in females than in males (25.0% vs. 18.6%), and in people ≥ 65 years than in people < 65 years (28.3% vs. 19.3%).

After adjusting for sex, education and deprivation, compared to low FIB4 scores, high FIB4 scores were cross-sectionally associated with higher prevalence of multimorbidity, with ORs of 1.13 (95%CI: 1.09, 1.16) for intermediate and 1.69 (1.55, 1.84) for high FIB4 score levels. Further adjustment for age significantly reduced the associations, with ORs decreased to1.04 (1.01, 1.08)) and 1.41 (1.30, 1.54), respectively. Additionally adjusting for lifestyle factors yielded similar association estimates, with ORs of 1.06 (1.03, 1.10) and 1.44 (1.32, 1.60). These associations were stronger in males than in females (OR for high vs. low FIB4 scores: 1.42 (1.29, 1.58) and 1.26 (1.08, 1.47) for males and females, relative OR (male to female) 1.32 (1.09, 1.59)). Supplementary Table 2 shows the results of subgroup analyses stratified by age, Townsend Deprivation Index, education, smoking, physical activity, BMI and diabetes. Most subgroups showed generally similar trends to the primary results. Although the association was not significant in some subgroups for high FIB4 scores, it was likely due to small sample sizes in these subgroups. Negative binomial regression revealed that intermediate and high FIB4 scores were associated with on 0.03 (0.02, 0.05) and 0.23 (0.19, 0.28) more LTCs than low FIB4 scores at baseline.

**Fig. 3:**
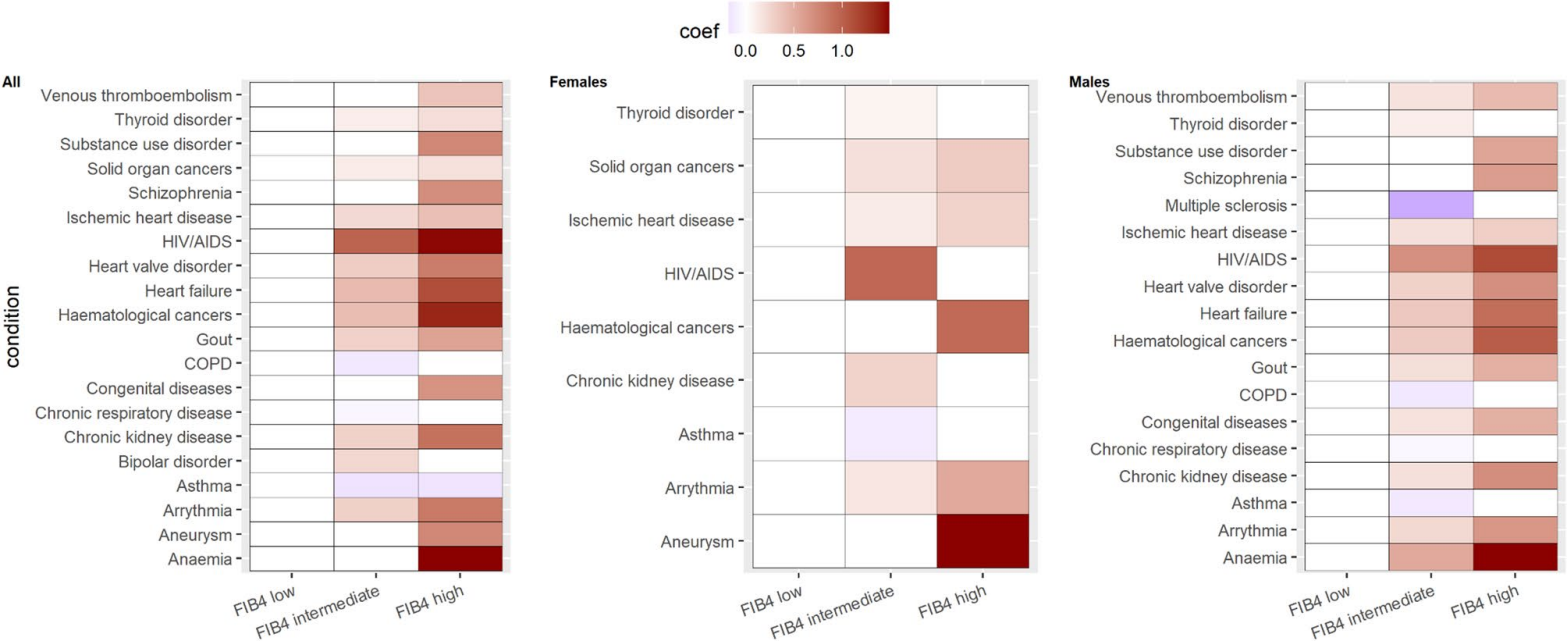
Heatmap showing the associations between baseline FIB4 score levels and prevalent long-term conditions in people with MASLD. Coefficients were estimated using logistic regression, with low FIB4 score level as reference. Red colour indicates higher prevalence, and blue lower prevalence. White colour indicates reference group (low FIB4 scores) or insignificant differences. The figure shows only the long-term conditions that showed significant differences for intermediate or high FIB4 score levels. FIB-4 score levels were defined using the cutoff values 1.30 and 2.67 to categorise low, intermediate and high levels for people < 65 years old, and 2.00 and 2.67 for people > = 65 years old

Supplementary Fig. 1 illustrates the relative ranks of LTCs by baseline prevalence across low, intermediate and high FIB4 score levels. Supplementary Table 3 provides the crude prevalences for these LTCs. Figure 3 and supplementary Table 4 show the associations between FIB4 score levels and LTC prevalences, overall and by sex. Overall, higher FIB4 scores were associated with lower prevalences of asthma and chronic obstructive pulmonary disease (COPD), but higher prevalences of CVDs (e.g., ischemic heart disease (IHD), heart valve disorder, heart failure, arrythmia and aneurysm), solid organ cancers, haematological cancers, chronic kidney disease (CKD), mental/behavioural conditions (e.g., substance use disorder, schizophrenia and bipolar disorders), HIV/AIDS, thyroid disorders, gout, congenital diseases and anaemia. High FIB4 scores were additionally associated with venous thromboembolism and multiple sclerosis in males.

Figure 1(B) shows the trajectory of multimorbidity prevalence during follow-up. Multimorbidity prevalence increased steadily by year across all FIB4 score groups. Low and intermediate FIB4 score groups had similar prevalence of multimorbidity during follow-up, substantially lower than high FIB4 score group, by approximately 10%. Specifically, the prevalence of multimorbidity in the low FIB4 group increased from 20.8% at baseline to 29.7%, 41.1% and 47.0% at year 5, 10, and 13. In the intermediate FIB4 group, prevalence rose from 21.8 to 31.5%, 43.6% and 50.8% at the same time points. In the high Fib4 group, it increased from 29.9 to 42.6%, 55.2% and 61.6%, respectively.

Among 61,579 people with 0 LTC at baseline, 22,141 (incidence 50.4%), 9225 (55.8%) and 696 (65.7%) people developed multimorbidity during a median follow-up of 12.7 (interquartile interval (IQI) 10.9, 13.8) years, in the low, intermediate and high FIB4 score groups; after adjusting for age, sex, education and socioeconomic status, the HR estimates were 0.99 (0.97, 1.02) and 1.09 (1.01, 1.18) for intermediate and high FIB4 scores, compared to low FIB4 scores.

All 38,679 people with 1 LTC at baseline developed multimorbidity during follow-up, with a median multimorbidity-free time of 10.3 (5.3, 12.1), 9.9 (4.9, 11.8) and 7.6 (3.4, 11.2) years for low, intermediate and high FIB4 score groups, respectively; the adjusted HR estimates were 0.96 (0.94, 1.00) and 1.08 (1.01, 1.15) for intermediate and high FIB4 scores, respectively.

### FIB4 scores, Multimorbidity and all-cause mortality

During a median follow-up of 13.7 years (IQI: 12.9, 14.4), 14,471 deaths (9379 males) were recorded. The median follow-up time was similar across the three FIB4 score levels: 13.8 (13.0, 14.5), 13.5 (12.7, 14.3) and 13.1 (12.5, 14.0) for low, intermediate and high FIB4 scores, respectively. Compared to people with low FIB4 scores, those with intermediate and high scores had a 45% (HR (95%CI) 1.45 (1.38, 1.51)) and 94% (1.94 (1.77, 2.13)) higher all-cause mortality, respectively. When additionally adjusted for multimorbidity, the HR estimates reduced by 9.0% to 1.40 (1.34, 1.46) for intermediate and by 10.0% to 1.82 (1.66, 2.00) for high FIB4 scores, respectively. Sensitivity analysis by excluding the first two years of follow-up showed similar results, with HR (95%CI) 1.43 (1.38, 1.48) and 1.91 (1.76, 2.07) reduced to 1.38 (1.33, 1.43) and 1.77 (1.63, 1.92) after adjustment for multimorbidity for intermediate and high FIB4 scores, respectively.

The association was stronger in females than in males. For intermediate FIB4 scores, the HRs were 1.50 (1.37, 1.63) in females and 1.36 (1.29, 1.44) in males, with a relative HR (RHR, male to female) of 0.89 (0.82, 0.96). For high FIB4 scores, the HRs were 2.21 (1.81, 2.70) in females and 1.71 (1.54, 1.90) in males, with an RHR of 0.76 (0.64, 0.91). (Table 3)

**Table 3:** Associations between baseline FIB4 score levels and all-cause mortality, with and without adjustment for Multimorbidity

		Events/total	HR (95%CI) *	HR (95%CI) with additional adjustment of multimorbidity	% reduction in coefficient estimates after adjustment**
Overall	FIB4 low	9517 / 90,035	Reference	Reference	
	FIB4 intermediate	4247 / 34,669	1.45 (1.38, 1.51)	1.40 (1.34, 1.46)	9.0
	FIB4 high	707 / 2766	1.94 (1.77, 2.13)	1.82 (1.66, 2.00)	10.0
Females	FIB4 low	3739 / 41,343	Reference	Reference	
	FIB4 intermediate	1180 / 11,002	1.53 (1.40, 1.66)	1.50 (1.37, 1.63)	4.5
	FIB4 high	173 / 748	2.33 (1.91, 2.84)	2.21 (1.81, 2.70)	6.2
Males	FIB4 low	5778 / 48,692	Reference	Reference	
	FIB4 intermediate	3067 / 23,667	1.42 (1.35, 1.50)	1.36 (1.29, 1.44)	11.1
	FIB4 high	534 / 2018	1.83 (1.65, 2.04)	1.71 (1.54, 1.90)	11.6

The Cox model was stratified by region and age group, adjusted for ethnicity, education, Townsend Deprivation Index (in fifths), physical activity, smoking status, and alcohol consumption. For the sex-mixed model, the model was additionally adjusted for sex. **: calculated as the (unadjusted coefficient– adjusted coefficient)*100/unadjusted coefficient. FIB-4 score levels were defined using the cutoff values 1.30 and 2.67 to categorise low, intermediate and high levels for people < 65 years old, and 2.00 and 2.67 for people > = 65 years old

We assessed the linear association between FIB4 score and all-cause mortality in a subset of participants after removing those with potential outlier FIB4 scores (> 99.5% quantile, *n* = 641), and found that each 1 unit increase in FIB4 score was associated with 44% higher mortality (1.44 (1.39, 1.48)), more pronounced in females (1.52 (1.44, 1.61)) than in males (1.39 (1.34, 1.45)).

Supplementary Table 5 shows the multimorbidity-adjusted associations between FIB4 scores and all-cause mortality subgroups stratified by age, Townsend Deprivation Index, education, smoking, physical activity, BMI levels, diabetes and multimorbidity status, which were similar to the primary results. (supplementary Table 5)

Table 4 shows the results of how additional adjustment for each individual LTC changed the association estimate between FIB4 scores and all-cause mortality, for selected LTCs, while supplementary Table 6 shows detailed results for all LTCs. The adjustment of CVDs (IHD, heart failure, stroke, heart valve disorder and arrythmia), solid organ cancers, haematological cancers, and CKD resulted in > 1% reduction in coefficient estimates. IHD, heart failure and arrythmia demonstrated the largest reduction. Adjusting for all these eight LTCs showed HR estimates of 1.34 (1.29, 1.39) and 1.73 (1.60, 1.87) for intermediate and high FIB4 score levels, respectively, corresponding to 21.2% and 17.3% reduction in association estimates. Adjusting for all 47 LTCs showed similar results.

**Table 4:** Associations between baseline FIB4 score level and all-cause mortality, with and without additional adjustment for long-term conditions (showing long term conditions with reduction in coefficient estimates > 1%)

	FIB4 Intermediate vs. low			FIB4 High vs. low	
	HR (95%CI)	% reduction in coefficient estimates		HR (95%CI)	% reduction in coefficient estimates
Without adjustment for long-term condition	1.45 (1.38, 1.51)	NA		1.94 (1.77, 2.13)	NA
**Additionally adjusted for the condition**:					
Ischemic heart disease	1.40 (1.34, 1.46)	9.5		1.85 (1.69, 2.03)	7.1
Heart failure	1.42 (1.36, 1.49)	4.2		1.87 (1.70, 2.05)	5.8
Stroke	1.44 (1.38, 1.51)	1.0		1.93 (1.76, 2.12)	1.1
Solid organ cancers	1.43 (1.36, 1.49)	4.0		1.92 (1.75, 2.11)	1.5
Haematological cancers	1.44 (1.38, 1.51)	0.9		1.91 (1.74, 2.10)	2.5
Chronic kidney disease	1.44 (1.37, 1.50)	1.7		1.92 (1.75, 2.10)	2.0
Heart valve disorder	1.44 (1.38, 1.50)	1.7		1.91 (1.74, 2.09)	2.7
Arrythmia	1.43 (1.36, 1.49)	4.0		1.87 (1.70, 2.05)	5.9
**Additionally adjusted for all 8 conditions above**	1.34 (1.29, 1.39)	21.2		1.73 (1.60, 1.87)	17.3
**Additionally adjusted for all 47 conditions**	1.34 (1.29, 1.39)	21.2		1.74 (1.61, 1.88)	17.3

FIB-4 score levels were defined using the cutoff values 1.30 and 2.67 to categorise low, intermediate and high levels for people < 65 years old, and 2.00 and 2.67 for people > = 65 years old

Using NAFLD fibrosis score for fibrosis assessment also showed positive associations with multimorbidity and mortality. The proportions of low, intermediate and high levels were 59,838 (46.9%), 58,056 (45.5%) and 9576 (7.5%), respectively. The association with multimorbidity was OR 1.23 (1.19, 1.30) and 1.82 (1.74, 1.90), the associations with mortality was HR 1.58 (1.53, 1.64) and 2.34 (2.22, 2.48) without MM adjustment, and 1.52 (1.46, 1.58) and 2.09 (1.98, 2.21) after multimorbidity adjustment, for intermediate and high levels, respectively.

## Discussion

In this cohort of 127,470 individuals with high risk of MASLD identified in UK Biobank, FIB4 scores were positively associated with the prevalence of multimorbidity, particularly with CVDs, extrahepatic cancers, CKD and mental/behavioural conditions. We observed a significant positive association between FIB4 scores and all-cause mortality, and adjustment for multimorbidity attenuated the association by 10%.

We found a positive link between FIB4 scores and prevalence and incidence of multimorbidity. A previous study reported that diabetic MASLD patients with advanced fibrosis stage (measured with transient elastography) had on average one more comorbidities (3.4 vs. 2.5), compared to those with lower fibrosis stages; however, this study had a small sample size of 82 individuals, considered only 15 LTCs to define multimorbidity, and only descriptively examined this association without adjusting for any confounders [23]. Our analysis highlighted the role of age in this association. Prior to adjusting for age, intermediate and high levels of FIB4 were associated with 13% and 69% higher risks of multimorbidity, respectively. However, adjusting for age reduced the associations: for intermediate FIB4 scores, the association became minimal, suggesting the association was mainly mediated by age; for high FIB4 scores, the OR reduced from 1.69 to 1.41, demonstrating age as a mediator. Among people ≥ 65 years old, both intermediate and high levels of FIB4 scores were associated with increased multimorbidity.

In our investigation on FIB4 scores and prevalence of specific LTCs, we found that higher FIB4 scores were positively associated with several conditions, including CVDs, solid organ cancers, haematological cancers, CKD, mental/behavioral diseases and thyroid disorder. The associations were observed in both males and females. While previous studies have reported associations between MASLD and these conditions [24–29], the relationships between fibrosis status and these conditions have been less clear. Prior research has indicated associations of liver fibrosis with CVDs [30] and CKD [31], but evidence regarding associations with extrahepatic cancers and mental/behavioral conditions remains inconclusive in people with MASLD [32].

The positive association between FIB4 scores and all-cause mortality in MASLD people was consistent with previous systematic reviews [6, 33]. In contrast, some previous UK Biobank investigations reported null evidence for such associations. For instance, Roca-Fernandez A. et al. found no significant association between FIB4 scores and all-cause mortality (HR 0.96 (0.80, 1.15)) in 33,616 UK Biobank participants with liver imaging data (27% with liver steatosis) with 2.5 year of follow-up [34]. Hydes T. et al. found that the association between FIB4 scores and mortality became null (HR 1.17 (0.94, 1.45)) after adjusting for smoking, diabetes, kidney function measures in 10,512 people with both MASLD and CKD [35]. These discrepancies may be attributed to their small sample size, short follow-up periods and the inclusion of participants with specific conditions in these studies.

This association between FIB4 scores and all-cause mortality was more pronounced in females than in males, which was consistent with a previous US study [13]. A systematic review and meta-analysis also identified sex as a significant contributor in the meta-regression, suggesting different underlying associations between males and females [6]. Previous research has observed sex difference in MASLD risk factor, progression and prognosis, with potential mechanisms involving difference in sociocultural gender difference, sex hormones, body fat distribution, lipid metabolism, glucose homeostasis, oxidate stress and inflammation, intestinal microbiome [36–38]. Moreover, our study also found women tend to have a higher burden of multimorbidity and may be disproportionately affected by complex care needs, fragmented healthcare delivery, and polypharmacy, all of which may interact with liver fibrosis to exacerbate mortality risk.

We also found that adjustment of multimorbidity reduced the associations between FIB4 and mortality, with a reduction of approximately 10% in the association estimates, higher in males than in females (11.1% vs. 4.5% for intermediate FIB4 scores, 11.6% vs. 6.2% for high scores). Notably, this reduction was mainly driven by extrahepatic cancers, CKD and CVDs. Among these conditions, IHD, heart failure, and arrythmia contributed the most to the mediation. These conditions have been previously linked to MASLD [24, 25, 27].

Although monitoring fibrosis progression has been one of the key focus in MASLD management [39], our findings suggest that multimorbidity may plays a role and should be emphasized in the management of MASLD. However, our analysis did not constitute a formal mediation analysis, and cannot support causal interpretation. These findings should be considered exploratory. Future research is warranted to clarify these relationships.

This study is the first large population-based cohort study to examine the relationship between liver fibrosis, multimorbidity and mortality, and it also provided exploratory evidence that multimorbidity may play a role in the association between fibrosis and mortality. This study’s strengths include a large sample size, long follow-up time, detailed phenotyping of multimorbidity, and robust sensitivity and subgroup analyses.

However, several limitations should be acknowledged. First, the study population was drawn from UK Biobank, which is predominantly white, less deprived, and healthier than the general UK population [40], therefore it may limit the generalisability of our results to other populations. Validation of our findings in independent, more representative cohorts is warranted to confirm their broader applicability. Second, we used FLI to assess steatosis, instead of biopsy histological data or accurate imaging techniques. Although FLI has been proven to be a valid surrogate for liver steatosis [12], we cannot exclude the potential misclassification bias introduced by using FLI rather than imaging or biopsy-confirmed steatosis. Third, we used FIB4 score as a non-invasive surrogate measurement for liver fibrosis stage, instead of the conventional gold standard of liver biopsy, which was not available. FIB4 has been demonstrated to be an accurate surrogate for fibrosis staging [41]. Sensitivity analyses using NAFLD fibrosis score confirmed the associations between fibrosis, multimorbidity and mortality. Fourth, we found that adjustment for multimorbidity reduced the HR estimates for FIB4 by 10%, however, this should not be interpreted as direct evidence for multimorbidity’s causal mediation effect, because this was a formal mediation analysis, thus this should be only interpreted descriptively. Future investigations using formal mediation analysis are warranted. Fifth, we did not account for the temporal sequence of LTCs, and the relatively small number of prevalent cases for certain conditions may also limited our statistical power. Sixth, we used a simple count-based definition of multimorbidity, which is commonly applied in population research. However, this approach does not account for differences in disease severity, duration, or impact. Alternative definitions, such as weighted comorbidity score or continuous measures, may offer additional insights and should be explored in future studies. Seventh, some variables were self-reported, including socioeconomic factors and lifestyle factors, which may introduce information bias. Specifically, self-reported alcohol consumption is subject to recall bias and underreporting. While objective biomarkers such as phosphatidylethanol (PEth) can provide more accurate assessments, these data are not available in UK Biobank, so we are unable to validate the self-reported alcohol intake against the biomarker. Eighth, we conducted complete-case analysis excluding participants with missing data required to calculate FIB-4 scores, which may have introduced selection bias. These exclusions may have disproportionately removed individuals with certain sociodemographic or clinical characteristics, potentially affecting the internal validity and generalizability of our findings. Future studies using imputation methods or sensitivity analyses could help assess the robustness of results under different assumptions about missingness. Ninth, although we adjusted for socioeconomic status and lifestyle factors in analyses, residual confounding remains due to the observational design of the study. Medication use and healthcare utilisation patterns are known risk factors for mortality. However, comprehensive data on medication use during follow-up are not available in the UK Biobank, which limits our ability to account for dynamic exposure to these medications over time. Additionally, we considered these medications to be potential mediators rather than confounders, as they lie on the causal pathway between liver fibrosis and adverse outcomes. Therefore, adjusting for them could introduce overadjustment bias and obscure the total effect of FIB4 scores on mortality. For these reasons, we did not include these variables in the main analyses.

## Conclusion

Our study highlighted that higher FIB4 scores, reflecting more advanced liver fibrosis in people with MASLD, were associated with higher prevalence of multimorbidity and higher all-cause mortality. Adjustment for multimorbidity attenuated the association between FIB-4 and all-cause mortality by approximately 10%. The association between FIB4 scores and all-cause mortality was stronger in females compared to males, highlighting potential sex differences in MASLD prognosis.

## Electronic supplementary material

Below is the link to the electronic supplementary material.

Supplementary Material 1

## Data Availability

UK Biobank data are available to registered researchers at https://www.ukbiobank.ac.uk/.

## Abbreviations

MASLD: metabolic dysfunction associated steatotic liver disease
LTC: long-term condition
FIB4: fibrosis-4 score
OR: odds ratio
HR: hazard ratio
CVD: cardiovascular disease
FLI: fatty liver index
BMI: body mass index
BP: blood pressure
TG: triglycerides
HDL: high density lipoprotein
HbA1c: glycated haemoglobin
MetALD: metabolic dysfunction and alcohol related liver disease
ALD: alcohol related liver disease
CMRF: cardiometabolic risk factor
SD: standard deviation
CI: confidence interval
