# Supplementary material for: Fibrosis status, extrahepatic multimorbidity and all-cause mortality in 53,093 women and 74,377 men with metabolic dysfunction associated steatotic liver disease (MASLD) in UK biobank
Source: BMC Gastroenterol. 2025 Jul 31;25:546. doi: 10.1186/s12876-025-04079-4 (PMC12315272; doi:10.1186/s12876-025-04079-4)
Supplement: Supplementary file 1 — Supplementary Material 1 [file 12876_2025_4079_MOESM1_ESM.docx]

Fibrosis status, extra-hepatic multimorbidity and mortality in 53093 women and 74377 men with MASLD in the UK Biobank

Supplementary materials

Table of Contents

[Supplementary methods 2](#_Toc188900420)

[Supplementary table 1: Baseline characteristics of participants with baseline MASLD by FIB-4 score levels (expanded). 3](#_Toc188900421)

[Supplementary table 2: Association between FIB4 scores and prevalence of multimorbidity (>= 2 conditions) in females and males with baseline MASLD 6](#_Toc188900422)

[Supplementary figure 1: Bump plot comparing the ranked long-term conditions based on crude prevalences in females and males with MASLD people by FIB-4 score level 9](#_Toc188900423)

[Supplementary table 3: Crude prevalences (per 1000) of long-term conditions in females and males with MASLD by FIB-4 score levels 10](#_Toc188900424)

[Supplementary table 4: Adjusted prevalence odds ratios (95% confidence intervals) comparing low, intermediate and high FIB-4 score levels on long-term conditions at baseline in people with baseline MASLD. 13](#_Toc188900425)

[Supplementary table 5: Subgroup analyses for the associations between baseline FIB4 score levels and all-cause mortality. 16](#_Toc188900426)

[Supplementary table 6: Associations between baseline FIB4 score levels and all-cause mortality, with and without additional adjustment for long-term conditions. 19](#_Toc188900427)

## Supplementary methods

The codelists for chronic liver diseases and long-term conditions for defining multimorbidity can be found online: [codelists.xlsx](https://docs.google.com/spreadsheets/d/1pErQT3krBj3ZpVk2JhM5styq_MYxzQOl/edit?usp=sharing&ouid=108654502587298592146&rtpof=true&sd=true)

## Supplementary table 1: Baseline characteristics of participants with baseline MASLD by FIB-4 score levels (expanded).

|  | FIB4 low | FIB4 intermediate | FIB4 high | Overall |
| --- | --- | --- | --- | --- |
|  | n = 90035 (70.6%) | n = 34668 (27.2%) | n = 2766 (2.2%) | n = 127470 (100.0%) |
| Sex, female | 41343 (45.9%) | 11001 (31.7%) | 748 (27.0%) | 53093 (41.7%) |
| **Age, years** | 56.6 (8.4) | 59.3 (5.6) | 62.9 (5.7) | 57.4 (7.9) |
| **Townsend deprivation index** |  |  |  |  |
| 1st fifth (least deprived) | 17698 (19.7%) | 7323 (21.1%) | 541 (19.6%) | 25562 (20.1%) |
| 2nd fifth | 17808 (19.8%) | 7098 (20.5%) | 585 (21.1%) | 25491 (20%) |
| 3rd fifth | 18014 (20.0%) | 6877 (19.8%) | 528 (19.1%) | 25419 (19.9%) |
| 4th fifth | 18072 (20.1%) | 6816 (19.7%) | 550 (19.9%) | 25438 (20%) |
| 5th fifth (most deprived) | 18328 (20.4%) | 6511 (18.8%) | 555 (20.1%) | 25394 (19.9%) |
| Missing | 115 (0.1%) | 44 (0.1%) | 7 (0.3%) | 166 (0.1%) |
| **Education** |  |  |  |  |
| Below secondary | 20653 (22.9%) | 8367 (24.1%) | 927 (33.5%) | 29947 (23.5%) |
| Lower secondary | 15182 (16.9%) | 5199 (15%) | 332 (12.0%) | 20713 (16.2%) |
| Higher secondary | 4555 (5.1%) | 1632 (4.7%) | 103 (3.7%) | 6290 (4.9%) |
| Vocational | 26211 (29.1%) | 10214 (29.5%) | 762 (27.5%) | 37187 (29.2%) |
| Higher education | 23365 (26%) | 9231 (26.6%) | 640 (23.1%) | 33236 (26.1%) |
| Missing | 69 (0.1%) | 26 (0.1%) | 2 (0.1%) | 97 (0.1%) |
| Ethnicity |  |  |  |  |
| White | 83828 (93.1%) | 32297 (93.2%) | 2579 (93.2%) | 118704 (93.1%) |
| Asian | 2587 (2.9%) | 777 (2.2%) | 59 (2.1%) | 3423 (2.7%) |
| Black | 1585 (1.8%) | 887 (2.6%) | 82 (3.0%) | 2554 (2.0%) |
| Others | 1581 (1.8%) | 530 (1.5%) | 30 (1.1%) | 2141 (1.7%) |
| Missing | 454 (0.5%) | 178 (0.5%) | 16 (0.6%) | 648 (0.5%) |
| **Smoking** |  |  |  |  |
| Never | 48103 (53.4%) | 17831 (51.4%) | 1327 (48.0%) | 67261 (52.8%) |
| Previous | 31946 (35.5%) | 13741 (39.6%) | 1196 (43.2%) | 46883 (36.8%) |
| Current | 9463 (10.5%) | 2879 (8.3%) | 217 (7.8%) | 12559 (9.9%) |
| Missing | 523 (0.6%) | 218 (0.6%) | 26 (0.9%) | 767 (0.6%) |
| **Alcohol drinking, units/week** | 7.9 (8.8) | 9.3 (9.2) | 9.2 (9.2) | 8.3 (8.9) |
| **Physical activity** |  |  |  |  |
| Low | 17185 (19.1%) | 5868 (16.9%) | 461 (16.7%) | 23514 (18.4%) |
| Moderate | 27172 (30.2%) | 10528 (30.4%) | 768 (27.8%) | 38468 (30.2%) |
| High | 22043 (24.5%) | 9696 (28.0%) | 797 (28.8%) | 32536 (25.5%) |
| Missing | 23635 (26.3%) | 8577 (24.7%) | 740 (26.8%) | 32952 (25.9%) |
| FIB4 score | 1.1 (0.3) | 1.7 (0.3) | 4.7 (20.3) | 1.3 (3.1) |
| BMI | 31.9 (4.7) | 31.4 (4.4) | 31.5 (4.6) | 31.8 (4.6) |
| **BMI** |  |  |  |  |
| < 25 | 1648 (1.8%) | 688 (2%) | 99 (3.6%) | 2435 (1.9%) |
| 25-30 | 33667 (37.4%) | 14329 (41.3%) | 1093 (39.5%) | 49089 (38.5%) |
| >30 | 54720 (60.8%) | 19652 (56.7%) | 1574 (56.9%) | 75946 (59.6%) |
| **Waist circumference, cm** | 102.4 (10.2) | 103.0 (10.1) | 104.2 (10.6) | 102.6 (10.2) |
| Waist-to-hip ratio, % | 92.7 (7.5) | 94.1 (7.3) | 95.3 (7.1) | 93.1 (7.5) |
| Waist-to-height ratio, % | 60.7 (6.2) | 60.2 (6.0) | 60.9 (6.4) | 60.6 (6.2) |
| Fat mass, kg | 32.6 (10.5) | 30.9 (9.8) | 30.7 (9.9) | 32.1 (10.3) |
| Fat-free mass, kg | 58.4 (11.3) | 61.2 (11.0) | 61.4 (10.7) | 59.2 (11.3) |
| Body fat percent, % | 35.6 (8.9) | 33.4 (8.4) | 33.1 (8.0) | 35.0 (8.8) |
| Systolic blood pressure, mmHg | 141.2 (17.4) | 142.5 (17.6) | 143.8 (18.6) | 141.6 (17.5) |
| Diastolic blood pressure, mmHg | 85.2 (9.7) | 85.0 (9.8) | 83.8 (10.3) | 85.1 (9.7) |
| Fasting time, hr | 3.8 (2.5) | 3.9 (2.3) | 4.0 (2.3) | 3.8 (2.4) |
| Habitual use of NSAID | 0.3 (0.5) | 0.3 (0.5) | 0.4 (0.5) | 0.3 (0.5) |
| Habitual use of statin | 0.1 (0.3) | 0.1 (0.3) | 0.1 (0.3) | 0.1 (0.3) |
| ALP, log_10_ U/L | 1.9 (0.1) | 1.9 (0.1) | 2.0 (0.2) | 1.9 (0.1) |
| ALT, log_10_ U/L | 1.4 (0.2) | 1.4 (0.2) | 1.5 (0.3) | 1.4 (0.2) |
| AST, log_10_ U/L | 1.4 (0.1) | 1.5 (0.1) | 1.6 (0.2) | 1.4 (0.1) |
| GGT, log_10_ U/L | 1.6 (0.3) | 1.6 (0.3) | 1.7 (0.4) | 1.6 (0.3) |
| Triglyceride, mmol/L | 2.2 [1.3] | 2.1 [1.3] | 2.0 [1.3] | 2.1 [1.3] |
| Cholesterol, mmol/L | 5.7 (1.2) | 5.5 (1.2) | 5.1 (1.3) | 5.7 (1.3) |
| HDL cholesterol, mmol/L | 1.1 (0.4) | 1.1 (0.4) | 1.1 (0.4) | 1.1 (0.4) |
| Direct LDL, mmol/L | 3.7 (0.9) | 3.5 (0.9) | 3.2 (0.9) | 3.6 (0.9) |
| Apolipoprotein A, g/L | 1.3 (0.4) | 1.3 (0.3) | 1.3 (0.4) | 1.3 (0.4) |
| Apolipoprotein B, g/L | 1.1 (0.3) | 1.0 (0.3) | 1.0 (0.3) | 1.1 (0.3) |
| Glucose, mmol/L | 5.0 (2.0) | 5.0 (1.9) | 5.3 (2.4) | 5.0 (2.0) |
| HbA1c, mmol/mol | 37.3 (10.1) | 36.8 (9.8) | 38.2 (11.9) | 37.2 (10.1) |
| CRP, mg/L | 2.4 [3.3] | 1.9 [2.6] | 2.1 [3.0] | 2.2 [3.1] |
| Vitamin D, nmol/L | 38.9 [28.3] | 42.6 [28.3] | 43.1 [30.0] | 40.0 [28.5] |
| Creatinine, umol/L | 73.0 [20.3] | 77.6 [20.1] | 79.2 [21.9] | 74.5 [20.5] |
| Total bilirubin, umol/L | 7.6 [3.8] | 8.6 [4.2] | 9.7 [5.4] | 7.9 [4.0] |
| Direct bilirubin, umol/L | 1.4 [0.8] | 1.6 [0.9] | 1.9 [1.2] | 1.4 [0.8] |
| Albumin, g/L | 42.7 (8.2) | 42.9 (8.0) | 42.3 (8.3) | 42.7 (8.1) |
| Urate, umol/L | 341.2 (75.9) | 355.2 (75.4) | 364.7 (84.8) | 345.5 (76.2) |
| Hemoglobin, g/dL | 14.4 (1.3) | 14.7 (1.2) | 14.6 (1.6) | 14.5 (1.3) |
| Red blood cell count, 10^12^ cells/L | 4.7 (0.4) | 4.7 (0.4) | 4.6 (0.5) | 4.7 (0.4) |
| Platelet, 10^9^ cells/L | 274.7 (56.8) | 209.8 (38.7) | 149.6 (51.6) | 254.4 (61.8) |
| White blood cell count, 10^9^ cells/L | 7.5 (1.9) | 6.9 (2.1) | 6.9 (4.4) | 7.3 (2.1) |
| Basophil cell count, 10^9^ cells/L | 0.0 [0.0] | 0.0 [0.0] | 0.0 [0.0] | 0.0 [0.0] |
| eosinophil cell count, 10^9^ cells/L | 0.2 [0.1] | 0.1 [0.1] | 0.1 [0.1] | 0.2 [0.1] |
| Lymphocyte count, 10^9^ cells/L | 2.0 [0.8] | 1.9 [0.8] | 1.8 [0.9] | 2.0 [0.8] |
| Monocyte count, 10^9^ cells/L | 0.5 [0.2] | 0.5 [0.2] | 0.5 [0.2] | 0.5 [0.2] |
| Hypertension | 72094 (80.1%) | 28023 (80.8%) | 2235 (80.8%) | 102352 (80.3%) |
| Obesity | 89089 (98.9%) | 34234 (98.7%) | 2706 (97.8%) | 126029 (98.9%) |
| Diabetes | 28339 (31.5%) | 10350 (29.9%) | 1047 (37.9%) | 39736 (31.2%) |
| High TG | 66192 (73.5%) | 24639 (71.1%) | 1788 (64.6%) | 92619 (72.7%) |
| Low HDL | 43112 (47.9%) | 14955 (43.1%) | 1323 (47.8%) | 59390 (46.6%) |

HbA1c: glycated hemoglobin. HDL: high density lipoprotein. LDL: low density lipoprotein. CRP: C-reactive protein. Number(number): mean(SD). Number(percent%): frequency(percent). Number[number]: median[interquartile range]. FIB-4 score levels were defined using the cutoff values 1.30 and 2.67 to categorise low, intermediate and high levels for people < 65 years old, and 2.00 and 2.67 for people >= 65 years old.

## Supplementary table 2: Association between FIB4 scores and prevalence of multimorbidity (>= 2 conditions) in females and males with baseline MASLD

|  |  | FIB4 low |  |  | FIB4 intermediate |  |  |  | FIB4 high |  |  |
| --- | --- | --- | --- | --- | --- | --- | --- | --- | --- | --- | --- |
|  | Subgroups | Prevalence | Prevalence OR |  | Prevalence | Prevalence OR | P value |  | Prevalence | Prevalence OR | P value |
| **Females** | Overall | 10186 / 41343 | 1 |  | 2876 / 11002 | 1.04 (0.99, 1.09) | 0.12 |  | 236 / 748 | 1.26 (1.08, 1.47) | <0.01 |
| Age | < 65 yrs | 7201 / 31130 | 1 |  | 2551 / 10070 | 1.06 (1.00, 1.12) | 0.05 |  | 103 / 314 | 1.16 (0.91, 1.47) | 0.23 |
|  | >= 65 yrs | 2985 / 10213 | 1 |  | 325 / 932 | 1.29 (1.12, 1.49) | <0.01 |  | 28 / 119 | 1.08 (0.70, 1.65) | 0.74 |
| Townsend deprivation index | Fifth 1 (least deprived) | 1544 / 7441 | 1 |  | 431 / 2031 | 0.99 (0.88, 1.12) | 0.87 |  | 46 / 142 | 1.60 (1.12, 2.28) | 0.01 |
|  | Fifth 2 | 1686 / 7825 | 1 |  | 473 / 2052 | 1.05 (0.93, 1.18) | 0.44 |  | 34 / 140 | 0.91 (0.61, 1.34) | 0.63 |
|  | Fifth 3 | 1945 / 8261 | 1 |  | 533 / 2131 | 1.03 (0.92, 1.15) | 0.58 |  | 63 / 166 | 1.51 (1.09, 2.07) | 0.01 |
|  | Fifth 4 | 2257 / 8670 | 1 |  | 677 / 2342 | 1.10 (0.99, 1.22) | 0.07 |  | 65 / 179 | 1.22 (0.90, 1.67) | 0.21 |
|  | Fifth 5 (most deprived) | 2742 / 9097 | 1 |  | 760 / 2431 | 1.03 (0.94, 1.14) | 0.53 |  | 98 / 279 | 1.21 (0.94, 1.55) | 0.14 |
| Education | Below secondary | 3090 / 10445 | 1 |  | 937 / 3150 | 1.03 (0.94, 1.12) | 0.54 |  | 38 / 136 | 1.26 (0.86, 1.84) | 0.24 |
|  | Lower secondary | 1861 / 8216 | 1 |  | 530 / 2121 | 1.10 (0.98, 1.23) | 0.1 |  | 7 / 29 | 0.90 (0.38, 2.15) | 0.82 |
|  | Higher secondary | 519 / 2173 | 1 |  | 113 / 528 | 0.80 (0.63, 1.02) | 0.07 |  | 56 / 179 | 1.33 (0.97, 1.84) | 0.08 |
|  | Vocational | 2703 / 11264 | 1 |  | 794 / 2994 | 1.09 (0.99, 1.20) | 0.07 |  | 37 / 125 | 1.37 (0.93, 2.03) | 0.11 |
|  | Higher education | 2006 / 9214 | 1 |  | 500 / 2200 | 0.99 (0.89, 1.11) | 0.93 |  | 125 / 420 | 1.35 (1.09, 1.67) | 0.01 |
| Smoking | Never | 5399 / 24266 | 1 |  | 1516 / 6346 | 1.06 (0.99, 1.13) | 0.07 |  | 84 / 269 | 1.05 (0.81, 1.37) | 0.71 |
|  | Previous | 3626 / 13106 | 1 |  | 1091 / 3758 | 1.03 (0.95, 1.11) | 0.52 |  | 25 / 54 | 1.95 (1.12, 3.37) | 0.02 |
|  | Current | 1103 / 3738 | 1 |  | 250 / 822 | 1.01 (0.85, 1.19) | 0.92 |  | 42 / 116 | 1.29 (0.88, 1.91) | 0.19 |
| Physical activity | Low | 2031 / 7517 | 1 |  | 531 / 1717 | 1.11 (0.99, 1.25) | 0.09 |  | 64 / 191 | 1.63 (1.20, 2.22) | <0.01 |
|  | Moderate | 2653 / 11976 | 1 |  | 728 / 3198 | 0.99 (0.90, 1.09) | 0.86 |  | 43 / 174 | 1.02 (0.71, 1.44) | 0.93 |
|  | High | 1917 / 8448 | 1 |  | 573 / 2378 | 1.04 (0.94, 1.16) | 0.43 |  | 11 / 26 | 2.44 (1.03, 5.79) | 0.04 |
| BMI | < 25.0 | 67 / 283 | 1 |  | 30 / 103 | 1.40 (0.82, 2.37) | 0.22 |  | 47 / 173 | 1.14 (0.81, 1.60) | 0.46 |
|  | 25.0-29.9 | 2150 / 9351 | 1 |  | 609 / 2631 | 1.00 (0.90, 1.11) | 0.99 |  | 178 / 549 | 1.25 (1.04, 1.50) | 0.02 |
|  | >= 30 | 7969 / 31709 | 1 |  | 2237 / 8268 | 1.04 (0.99, 1.10) | 0.13 |  | 133 / 434 | 1.35 (1.10, 1.67) | <0.01 |
| **diabetes** | No diabetes | 14337 / 27215 | 1 |  | 4164 / 7335 | 1.02 (0.96, 1.07) | 0.53 |  | 286 / 434 | 1.32 (1.07, 1.62) | <0.01 |
|  | Diabetes | 9093 / 14128 | 1 |  | 2381 / 3667 | 0.96 (0.89, 1.04) | 0.37 |  | 245 / 314 | 1.65 (1.25, 2.17) | <0.01 |
|  |  |  |  |  |  |  |  |  |  |  |  |
| **Males** | Overall | 8545 / 48692 | 1 |  | 4667 / 23667 | 1.04 (1.00, 1.09) | 0.04 |  | 590 / 2018 | 1.42 (1.29, 1.58) | < 0.01 |
| Age | < 65 yrs | 5320 / 36445 | 1 |  | 3857 / 20863 | 1.08 (1.02, 1.13) | <0.01 |  | 241 / 979 | 1.47 (1.26, 1.71) | <0.01 |
|  | >= 65 yrs | 3225 / 12247 | 1 |  | 810 / 2804 | 1.14 (1.04, 1.25) | 0.01 |  | 349 / 1039 | 1.39 (1.21, 1.59) | <0.01 |
| Townsend deprivation index | Fifth 1 (least deprived) | 1466 / 10257 | 1 |  | 870 / 5292 | 1.06 (0.97, 1.16) | 0.22 |  | 114 / 422 | 1.62 (1.29, 2.03) | <0.01 |
|  | Fifth 2 | 1542 / 9983 | 1 |  | 909 / 5046 | 1.08 (0.99, 1.19) | 0.08 |  | 130 / 443 | 1.63 (1.32, 2.02) | <0.01 |
|  | Fifth 3 | 1577 / 9753 | 1 |  | 899 / 4746 | 1.09 (0.99, 1.20) | 0.06 |  | 99 / 388 | 1.32 (1.04, 1.68) | 0.02 |
|  | Fifth 4 | 1784 / 9402 | 1 |  | 923 / 4474 | 0.98 (0.89, 1.07) | 0.61 |  | 108 / 384 | 1.19 (0.94, 1.50) | 0.15 |
|  | Fifth 5 (most deprived) | 2163 / 9231 | 1 |  | 1062 / 4080 | 1.03 (0.94, 1.12) | 0.57 |  | 137 / 376 | 1.41 (1.13, 1.76) | <0.01 |
| Education | Below secondary | 2611 / 10208 | 1 |  | 1442 / 5217 | 1.11 (1.03, 1.20) | 0.01 |  | 220 / 648 | 1.32 (1.11, 1.57) | <0.01 |
|  | Lower secondary | 1137 / 6966 | 1 |  | 593 / 3078 | 1.06 (0.94, 1.18) | 0.33 |  | 55 / 196 | 1.51 (1.09, 2.10) | 0.01 |
|  | Higher secondary | 382 / 2382 | 1 |  | 165 / 1104 | 0.79 (0.65, 0.97) | 0.02 |  | 22 / 74 | 1.47 (0.86, 2.51) | 0.15 |
|  | Vocational | 2412 / 14947 | 1 |  | 1397 / 7220 | 1.10 (1.02, 1.18) | 0.01 |  | 159 / 583 | 1.44 (1.18, 1.74) | <0.01 |
|  | Higher education | 1995 / 14151 | 1 |  | 1069 / 7031 | 0.95 (0.87, 1.03) | 0.19 |  | 134 / 515 | 1.58 (1.28, 1.94) | <0.01 |
| Smoking | Never | 3299 / 23837 | 1 |  | 1889 / 11485 | 1.07 (1.01, 1.14) | 0.03 |  | 234 / 907 | 1.57 (1.34, 1.84) | <0.01 |
|  | Previous | 4051 / 18840 | 1 |  | 2292 / 9983 | 1.04 (0.98, 1.11) | 0.15 |  | 309 / 927 | 1.40 (1.21, 1.62) | <0.01 |
|  | Current | 1116 / 5725 | 1 |  | 463 / 2057 | 1.11 (0.97, 1.26) | 0.12 |  | 39 / 163 | 1.10 (0.76, 1.61) | 0.62 |
| Physical activity | Low | 1941 / 9668 | 1 |  | 954 / 4151 | 1.04 (0.95, 1.14) | 0.38 |  | 115 / 345 | 1.43 (1.13, 1.82) | <0.01 |
|  | Moderate | 2486 / 15196 | 1 |  | 1392 / 7330 | 1.07 (0.99, 1.16) | 0.06 |  | 156 / 577 | 1.41 (1.16, 1.71) | <0.01 |
|  | High | 2037 / 13595 | 1 |  | 1211 / 7318 | 1.02 (0.94, 1.10) | 0.63 |  | 166 / 623 | 1.50 (1.24, 1.81) | <0.01 |
| BMI | < 25.0 | 216 / 1365 | 1 |  | 109 / 585 | 1.19 (0.92, 1.55) | 0.18 |  | 23 / 73 | 2.20 (1.29, 3.75) | <0.01 |
|  | 25.0-29.9 | 3667 / 24316 | 1 |  | 1912 / 11698 | 1.00 (0.94, 1.07) | 0.90 |  | 253 / 920 | 1.60 (1.37, 1.86) | <0.01 |
|  | >= 30 | 4662 / 23011 | 1 |  | 2646 / 11384 | 1.06 (1.01, 1.12) | 0.03 |  | 314 / 1025 | 1.23 (1.07, 1.42) | <0.01 |
| Diabetes | No diabetes | 15048 / 34481 | 1 |  | 8691 / 16984 | 1.02 (0.98, 1.06) | 0.36 |  | 842 / 1285 | 1.37 (1.21, 1.54) | <0.01 |
|  | diabetes | 8943 / 14211 | 1 |  | 4318 / 6683 | 0.96 (0.90, 1.02) | 0.22 |  | 554 / 733 | 1.28 (1.07, 1.53) | <0.01 |

OR was estimated using logistic regression, adjusted for age, ethnicity, education and Townsend Deprivation Index (in fifths). FIB-4 score levels were defined using the cutoff values 1.30 and 2.67 to categorise low, intermediate and high levels for people < 65 years old, and 2.00 and 2.67 for people >= 65 years old.


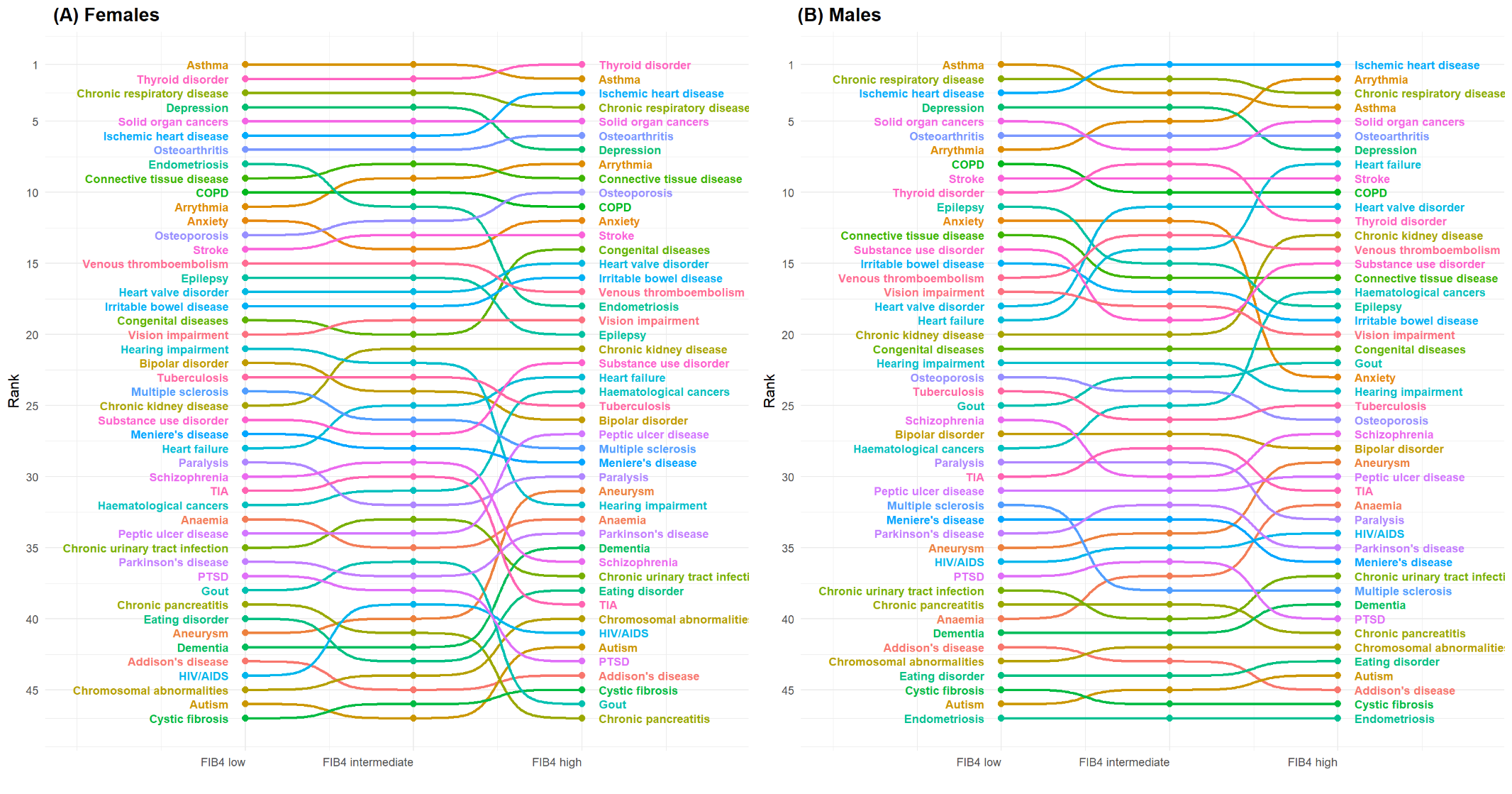


## Supplementary figure 1: Bump plot comparing the ranked long-term conditions based on crude prevalences in females and males with MASLD people by FIB-4 score level

FIB-4 score levels were defined using the cutoff values 1.30 and 2.67 to categorise low, intermediate and high levels for people < 65 years old, and 2.00 and 2.67 for people >= 65 years old.

## Supplementary table 3: Crude prevalences (per 1000) of long-term conditions in females and males with MASLD by FIB-4 score levels

|  | Females |  |  |  | Males |  |  |
| --- | --- | --- | --- | --- | --- | --- | --- |
| Rank | FIB4 low | FIB4 intermediate | FIB4 high |  | FIB4 low | FIB4 intermediate | FIB4 high |
| 1 | Asthma (159.3) | Asthma (135.6) | Thyroid disorder (164.4) |  | Asthma (116.0) | Ischemic heart disease (123.6) | Ischemic heart disease (194.7) |
| 2 | Thyroid disorder (117.8) | Thyroid disorder (134.3) | Asthma (137.7) |  | Chronic respiratory disease (114.5) | Chronic respiratory disease (105.1) | Arrythmia (113.0) |
| 3 | Chronic respiratory disease (100.7) | Chronic respiratory disease (94.8) | Ischemic heart disease (107.0) |  | Ischemic heart disease (88.7) | Asthma (95.5) | Chronic respiratory disease (99.1) |
| 4 | Depression (98.1) | Depression (90.9) | Chronic respiratory disease (107.0) |  | Depression (52.9) | Depression (51.5) | Asthma (92.7) |
| 5 | Solid organ cancers (57.6) | Solid organ cancers (78.3) | Solid organ cancers (104.3) |  | Solid organ cancers (38.2) | Arrythmia (49.8) | Solid organ cancers (60.5) |
| 6 | Ischemic heart disease (53.1) | Ischemic heart disease (67.4) | Osteoarthritis (77.5) |  | Osteoarthritis (36.6) | Osteoarthritis (44.0) | Osteoarthritis (55.5) |
| 7 | Osteoarthritis (50.8) | Osteoarthritis (55.1) | Depression (69.5) |  | Arrythmia (32.6) | Solid organ cancers (43.7) | Depression (48.1) |
| 8 | Endometriosis (31.0) | Connective tissue disease (32.4) | Arrythmia (58.8) |  | COPD (24.1) | Thyroid disorder (26.6) | Heart failure (41.1) |
| 9 | Connective tissue disease (28.2) | Arrythmia (29.7) | Connective tissue disease (38.8) |  | Stroke (23.5) | Stroke (24.1) | Stroke (39.6) |
| 10 | COPD (24.4) | COPD (25.2) | Osteoporosis (36.1) |  | Thyroid disorder (21.3) | COPD (22.0) | COPD (36.7) |
| 11 | Arrythmia (22.4) | Endometriosis (24.7) | COPD (34.8) |  | Epilepsy (13.3) | Heart valve disorder (15.5) | Heart valve disorder (33.7) |
| 12 | Anxiety (21.6) | Osteoporosis (24.2) | Anxiety (26.7) |  | Anxiety (13.3) | Anxiety (14.1) | Thyroid disorder (29.2) |
| 13 | Osteoporosis (20.6) | Stroke (22.4) | Stroke (26.7) |  | Connective tissue disease (11.5) | Venous thromboembolism (14.0) | Chronic kidney disease (24.8) |
| 14 | Stroke (18.3) | Anxiety (19.1) | Congenital diseases (17.4) |  | Substance use disorder (10.6) | Heart failure (13.9) | Venous thromboembolism (23.3) |
| 15 | Venous thromboembolism (14.9) | Venous thromboembolism (15.4) | Heart valve disorder (17.4) |  | Irritable bowel disease (10.3) | Epilepsy (12.6) | Substance use disorder (22.3) |
| 16 | Epilepsy (12.4) | Epilepsy (14.0) | Irritable bowel disease (16.0) |  | Venous thromboembolism (10.2) | Connective tissue disease (12.3) | Connective tissue disease (19.8) |
| 17 | Heart valve disorder (10.6) | Heart valve disorder (13.7) | Venous thromboembolism (14.7) |  | Vision impairment (9.8) | Irritable bowel disease (10.9) | Haematological cancers (16.8) |
| 18 | Irritable bowel disease (10.5) | Irritable bowel disease (11.7) | Endometriosis (14.7) |  | Heart valve disorder (9.7) | Vision impairment (10.8) | Epilepsy (16.4) |
| 19 | Congenital diseases (9.2) | Vision impairment (9.6) | Vision impairment (12.0) |  | Heart failure (8.1) | Substance use disorder (10.7) | Irritable bowel disease (14.9) |
| 20 | Vision impairment (8.5) | Congenital diseases (8.5) | Epilepsy (10.7) |  | Chronic kidney disease (7.3) | Chronic kidney disease (10.3) | Vision impairment (13.9) |
| 21 | Hearing impairment (5.8) | Chronic kidney disease (7.4) | Chronic kidney disease (10.7) |  | Congenital diseases (7.3) | Congenital diseases (8.9) | Congenital diseases (13.4) |
| 22 | Bipolar disorder (5.7) | Hearing impairment (7.4) | Substance use disorder (9.4) |  | Hearing impairment (6.5) | Hearing impairment (6.9) | Gout (11.9) |
| 23 | Tuberculosis (5.5) | Tuberculosis (7.3) | Heart failure (9.4) |  | Osteoporosis (5.7) | Gout (6.3) | Anxiety (10.4) |
| 24 | Multiple sclerosis (4.9) | Bipolar disorder (6.7) | Haematological cancers (9.4) |  | Tuberculosis (4.6) | Osteoporosis (6.2) | Hearing impairment (7.9) |
| 25 | Chronic kidney disease (4.8) | Heart failure (6.1) | Tuberculosis (8.0) |  | Gout (4.4) | Haematological cancers (5.8) | Tuberculosis (6.4) |
| 26 | Substance use disorder (4.6) | Multiple sclerosis (5.3) | Bipolar disorder (8.0) |  | Schizophrenia (3.8) | Tuberculosis (4.7) | Osteoporosis (6.4) |
| 27 | Meniere's disease (4.3) | Substance use disorder (4.8) | Peptic ulcer disease (8.0) |  | Bipolar disorder (3.8) | Bipolar disorder (4.4) | Schizophrenia (5.5) |
| 28 | Heart failure (3.9) | Meniere's disease (4.8) | Multiple sclerosis (6.7) |  | Haematological cancers (3.5) | TIA (4.0) | Bipolar disorder (5.0) |
| 29 | Paralysis (3.4) | Schizophrenia (3.7) | Meniere's disease (6.7) |  | Paralysis (3.5) | Paralysis (3.8) | Aneurysm (5.0) |
| 30 | Schizophrenia (3.1) | TIA (3.7) | Paralysis (5.3) |  | TIA (3.3) | Schizophrenia (3.2) | Peptic ulcer disease (5.0) |
| 31 | TIA (2.6) | Haematological cancers (3.7) | Aneurysm (5.3) |  | Peptic ulcer disease (3.0) | Peptic ulcer disease (2.8) | TIA (4.0) |
| 32 | Haematological cancers (2.6) | Paralysis (3.5) | Hearing impairment (5.3) |  | Multiple sclerosis (2.5) | Parkinson's disease (2.6) | Anaemia (4.0) |
| 33 | Anaemia (2.2) | Chronic urinary tract infection (2.3) | Anaemia (4.0) |  | Meniere's disease (2.5) | Meniere's disease (2.5) | Paralysis (3.5) |
| 34 | Peptic ulcer disease (1.9) | Peptic ulcer disease (2.3) | Parkinson's disease (2.7) |  | Parkinson's disease (1.9) | Aneurysm (2.2) | HIV/AIDS (3.0) |
| 35 | Chronic urinary tract infection (1.5) | Anaemia (2.1) | Dementia (2.7) |  | Aneurysm (1.8) | HIV/AIDS (2.2) | Parkinson's disease (3.0) |
| 36 | Parkinson's disease (1.3) | Gout (1.5) | Schizophrenia (2.7) |  | HIV/AIDS (1.3) | PTSD (1.4) | Meniere's disease (2.0) |
| 37 | PTSD (1.1) | Parkinson's disease (1.2) | Chronic urinary tract infection (1.3) |  | PTSD (1.1) | Anaemia (1.3) | Chronic urinary tract infection (1.5) |
| 38 | Gout (0.9) | PTSD (1.0) | Eating disorder (1.3) |  | Chronic urinary tract infection (0.9) | Multiple sclerosis (1.1) | Multiple sclerosis (1.5) |
| 39 | Chronic pancreatitis (0.6) | HIV/AIDS (0.7) | TIA (1.3) |  | Chronic pancreatitis (0.8) | Chronic pancreatitis (1.1) | Dementia (1.0) |
| 40 | Eating disorder (0.6) | Aneurysm (0.6) | Chromosomal abnormalities (0.0) |  | Anaemia (0.7) | Chronic urinary tract infection (0.9) | PTSD (1.0) |
| 41 | Aneurysm (0.6) | Chronic pancreatitis (0.5) | HIV/AIDS (0.0) |  | Dementia (0.5) | Dementia (0.7) | Chronic pancreatitis (0.5) |
| 42 | Dementia (0.5) | Dementia (0.4) | Autism (0.0) |  | Addison's disease (0.2) | Chromosomal abnormalities (0.2) | Chromosomal abnormalities (0.0) |
| 43 | Addison's disease (0.5) | Eating disorder (0.4) | PTSD (0.0) |  | Chromosomal abnormalities (0.1) | Addison's disease (0.2) | Eating disorder (0.0) |
| 44 | HIV/AIDS (0.4) | Chromosomal abnormalities (0.2) | Addison's disease (0.0) |  | Eating disorder (0.1) | Eating disorder (0.0) | Autism (0.0) |
| 45 | Chromosomal abnormalities (0.2) | Addison's disease (0.2) | Cystic fibrosis (0.0) |  | Cystic fibrosis (0.1) | Autism (0.0) | Addison's disease (0.0) |
| 46 | Autism (0.1) | Cystic fibrosis (0.1) | Gout (0.0) |  | Autism (0.0) | Cystic fibrosis (0.0) | Cystic fibrosis (0.0) |
| 47 | Cystic fibrosis (0.0) | Autism (0.0) | Chronic pancreatitis (0.0) |  | Endometriosis (0.0) | Endometriosis (0.0) | Endometriosis (0.0) |

Showing the condition (prevalence, /1000) at baseline. The conditions were ranked by prevalences. MASLD: metabolic dysfunction-associated steatotic liver disease. HIV: human immunodeficiency virus. PTSD: post-trauma stress disorder. IHD: ischemic heart disease. TIA: transient ischemic attach. COPD: chronic obstructive pulmonary disease. IBD: inflammatory bowel disease. VTE: venous thromboembolism. FIB-4 score levels were defined using the cutoff values 1.30 and 2.67 to categorise low, intermediate and high levels for people < 65 years old, and 2.00 and 2.67 for people >= 65 years old.

## Supplementary table 4: Adjusted prevalence odds ratios (95% confidence intervals) comparing low, intermediate and high FIB-4 score levels on long-term conditions at baseline in people with baseline MASLD.

|  | FIB4 low |  |  | FIB4 intermediate |  |  |  | FIB4 high |  |  |
| --- | --- | --- | --- | --- | --- | --- | --- | --- | --- | --- |
| condition | Prevalence | Prevalence OR |  | Prevalence | Prevalence OR | P value* |  | Prevalence | Prevalence OR | P value* |
| **All** |  |  |  |  |  |  |  |  |  |  |
| Congenital diseases | 733 / 90035 | 1 |  | 304 / 34669 | 1.14 (0.99, 1.31) | 0.15 |  | 40 / 2766 | 1.97 (1.42, 2.73) | <0.01 |
| HIV/AIDS | 78 / 90035 | 1 |  | 60 / 34669 | 2.68 (1.87, 3.85) | <0.01 |  | 6 / 2766 | 4.36 (1.84, 10.28) | <0.01 |
| Schizophrenia | 315 / 90035 | 1 |  | 117 / 34669 | 1.25 (0.99, 1.57) | 0.14 |  | 13 / 2766 | 2.05 (1.16, 3.61) | 0.04 |
| Bipolar disorder | 422 / 90035 | 1 |  | 179 / 34669 | 1.28 (1.07, 1.54) | 0.03 |  | 16 / 2766 | 1.62 (0.98, 2.69) | 0.15 |
| Substance use disorder | 707 / 90035 | 1 |  | 306 / 34669 | 1.07 (0.93, 1.23) | 0.54 |  | 52 / 2766 | 2.13 (1.59, 2.85) | <0.01 |
| Ischemic heart disease | 6512 / 90035 | 1 |  | 3667 / 34669 | 1.26 (1.21, 1.32) | <0.01 |  | 473 / 2766 | 1.48 (1.33, 1.65) | <0.01 |
| Heart failure | 554 / 90035 | 1 |  | 397 / 34669 | 1.54 (1.35, 1.75) | <0.01 |  | 90 / 2766 | 3.09 (2.45, 3.89) | <0.01 |
| COPD | 2181 / 90035 | 1 |  | 798 / 34669 | 0.87 (0.80, 0.94) | <0.01 |  | 100 / 2766 | 1.03 (0.84, 1.27) | 0.99 |
| Chronic respiratory disease | 9736 / 90035 | 1 |  | 3530 / 34669 | 0.95 (0.91, 0.99) | 0.03 |  | 280 / 2766 | 0.98 (0.86, 1.11) | 0.99 |
| Asthma | 12237 / 90035 | 1 |  | 3753 / 34669 | 0.84 (0.81, 0.87) | <0.01 |  | 290 / 2766 | 0.85 (0.75, 0.96) | 0.03 |
| Solid organ cancers | 4244 / 90035 | 1 |  | 1896 / 34669 | 1.13 (1.06, 1.19) | <0.01 |  | 200 / 2766 | 1.20 (1.04, 1.40) | 0.04 |
| Haematological cancers | 276 / 90035 | 1 |  | 179 / 34669 | 1.51 (1.25, 1.83) | <0.01 |  | 41 / 2766 | 3.88 (2.77, 5.45) | <0.01 |
| Chronic kidney disease | 555 / 90035 | 1 |  | 324 / 34669 | 1.32 (1.15, 1.52) | <0.01 |  | 58 / 2766 | 2.45 (1.86, 3.24) | <0.01 |
| Heart valve disorder | 910 / 90035 | 1 |  | 517 / 34669 | 1.36 (1.22, 1.52) | <0.01 |  | 81 / 2766 | 2.28 (1.80, 2.88) | <0.01 |
| Arrythmia | 2512 / 90035 | 1 |  | 1505 / 34669 | 1.33 (1.25, 1.42) | <0.01 |  | 272 / 2766 | 2.32 (2.03, 2.66) | <0.01 |
| Venous thromboembolism | 1111 / 90035 | 1 |  | 500 / 34669 | 1.13 (1.02, 1.26) | 0.07 |  | 58 / 2766 | 1.44 (1.10, 1.89) | 0.03 |
| Aneurysm | 111 / 90035 | 1 |  | 60 / 34669 | 1.13 (0.82, 1.55) | 0.61 |  | 14 / 2766 | 2.12 (1.20, 3.72) | 0.03 |
| Thyroid disorder | 5904 / 90035 | 1 |  | 2108 / 34669 | 1.12 (1.06, 1.18) | <0.01 |  | 182 / 2766 | 1.22 (1.04, 1.43) | 0.04 |
| Gout | 250 / 90035 | 1 |  | 166 / 34669 | 1.33 (1.09, 1.62) | 0.02 |  | 24 / 2766 | 1.78 (1.16, 2.73) | 0.03 |
| Anaemia | 124 / 90035 | 1 |  | 53 / 34669 | 1.46 (1.04, 2.04) | 0.08 |  | 11 / 2766 | 4.44 (2.35, 8.39) | <0.01 |
|  |  |  |  |  |  |  |  |  |  |  |
| **Females** |  |  |  |  |  |  |  |  |  |  |
| HIV/AIDS | 17 / 41343 | 1 |  | 8 / 11002 | 3.58 (1.43, 8.92) | 0.04 |  | 0 / 748 | NA | NA |
| Ischemic heart disease | 2194 / 41343 | 1 |  | 742 / 11002 | 1.18 (1.08, 1.29) | <0.01 |  | 80 / 748 | 1.45 (1.14, 1.84) | 0.03 |
| Asthma | 6587 / 41343 | 1 |  | 1492 / 11002 | 0.85 (0.79, 0.90) | <0.01 |  | 103 / 748 | 0.87 (0.71, 1.08) | 0.47 |
| Solid organ cancers | 2382 / 41343 | 1 |  | 861 / 11002 | 1.28 (1.18, 1.39) | <0.01 |  | 78 / 748 | 1.53 (1.20, 1.94) | 0.01 |
| Haematological cancers | 106 / 41343 | 1 |  | 41 / 11002 | 1.42 (0.99, 2.05) | 0.20 |  | 7 / 748 | 3.49 (1.61, 7.59) | 0.02 |
| Chronic kidney disease | 198 / 41343 | 1 |  | 81 / 11002 | 1.44 (1.11, 1.87) | 0.04 |  | 8 / 748 | 1.84 (0.90, 3.77) | 0.40 |
| Arrythmia | 925 / 41343 | 1 |  | 327 / 11002 | 1.23 (1.08, 1.40) | 0.02 |  | 44 / 748 | 2.04 (1.49, 2.79) | <0.01 |
| Aneurysm | 24 / 41343 | 1 |  | 7 / 11002 | 1.00 (0.43, 2.32) | 0.99 |  | 4 / 748 | 7.19 (2.44, 21.19) | 0.01 |
| Thyroid disorder | 4869 / 41343 | 1 |  | 1478 / 11002 | 1.10 (1.03, 1.17) | 0.03 |  | 123 / 748 | 1.29 (1.06, 1.57) | 0.07 |
|  |  |  |  |  |  |  |  |  |  |  |
| **Males** |  |  |  |  |  |  |  |  |  |  |
| Congenital diseases | 354 / 48692 | 1 |  | 211 / 23667 | 1.27 (1.06, 1.51) | 0.03 |  | 27 / 2018 | 1.94 (1.30, 2.9) | 0.01 |
| HIV/AIDS | 61 / 48692 | 1 |  | 52 / 23667 | 2.54 (1.71, 3.77) | <0.01 |  | 6 / 2018 | 4.60 (1.92, 11.00) | <0.01 |
| Schizophrenia | 186 / 48692 | 1 |  | 76 / 23667 | 1.11 (0.84, 1.47) | 0.68 |  | 11 / 2018 | 2.27 (1.21, 4.25) | 0.04 |
| Substance use disorder | 516 / 48692 | 1 |  | 253 / 23667 | 1.08 (0.92, 1.26) | 0.63 |  | 45 / 2018 | 2.13 (1.55, 2.92) | <0.01 |
| Ischemic heart disease | 4318 / 48692 | 1 |  | 2925 / 23667 | 1.29 (1.22, 1.36) | <0.01 |  | 393 / 2018 | 1.49 (1.32, 1.68) | <0.01 |
| Heart failure | 393 / 48692 | 1 |  | 330 / 23667 | 1.58 (1.36, 1.83) | <0.01 |  | 83 / 2018 | 3.42 (2.67, 4.38) | <0.01 |
| COPD | 1174 / 48692 | 1 |  | 521 / 23667 | 0.83 (0.75, 0.92) | <0.01 |  | 74 / 2018 | 0.98 (0.77, 1.25) | 0.99 |
| Chronic respiratory disease | 5574 / 48692 | 1 |  | 2487 / 23667 | 0.93 (0.89, 0.98) | 0.03 |  | 200 / 2018 | 0.92 (0.79, 1.06) | 0.57 |
| Asthma | 5650 / 48692 | 1 |  | 2261 / 23667 | 0.83 (0.79, 0.88) | <0.01 |  | 187 / 2018 | 0.83 (0.71, 0.97) | 0.07 |
| Haematological cancers | 170 / 48692 | 1 |  | 138 / 23667 | 1.55 (1.23, 1.94) | <0.01 |  | 34 / 2018 | 3.94 (2.69, 5.76) | <0.01 |
| Multiple sclerosis | 124 / 48692 | 1 |  | 27 / 23667 | 0.49 (0.32, 0.75) | <0.01 |  | 3 / 2018 | 0.70 (0.22, 2.22) | 0.92 |
| Chronic kidney disease | 357 / 48692 | 1 |  | 243 / 23667 | 1.29 (1.09, 1.52) | 0.01 |  | 50 / 2018 | 2.58 (1.90, 3.50) | <0.01 |
| Heart valve disorder | 470 / 48692 | 1 |  | 366 / 23667 | 1.45 (1.26, 1.66) | <0.01 |  | 68 / 2018 | 2.56 (1.97, 3.33) | <0.01 |
| Arrythmia | 1587 / 48692 | 1 |  | 1178 / 23667 | 1.37 (1.27, 1.48) | <0.01 |  | 228 / 2018 | 2.39 (2.06, 2.77) | <0.01 |
| Venous thromboembolism | 495 / 48692 | 1 |  | 331 / 23667 | 1.26 (1.10, 1.46) | 0.01 |  | 47 / 2018 | 1.76 (1.30, 2.39) | <0.01 |
| Thyroid disorder | 1035 / 48692 | 1 |  | 630 / 23667 | 1.16 (1.04, 1.28) | 0.02 |  | 59 / 2018 | 1.10 (0.84, 1.44) | 0.86 |
| Gout | 213 / 48692 | 1 |  | 149 / 23667 | 1.30 (1.05, 1.61) | 0.04 |  | 24 / 2018 | 1.93 (1.25, 2.96) | 0.01 |
| Anaemia | 34 / 48692 | 1 |  | 30 / 23667 | 2.05 (1.23, 3.41) | 0.02 |  | 8 / 2018 | 7.28 (3.22, 16.43) | <0.01 |

* P values were corrected for multiple comparisons using the false discovery rate (FDR) method.

The OR (95%CI)s were adjusted for sex, age, education level and Townsend Deprivation Index (in fifth), representing the excessive risk of having a specific long-term condition in people with intermediate or high FIB4 scores, compared to people with low FIB4 scores.

HIV: human immunodeficiency virus. IHD: ischemic heart disease. TIA: transient ischemic attach. COPD: chronic obstructive pulmonary disease. VTE: venous thromboembolism. FIB-4 score levels were defined using the cutoff values 1.30 and 2.67 to categorise low, intermediate and high levels for people < 65 years old, and 2.00 and 2.67 for people >= 65 years old.

## Supplementary table 5: Subgroup analyses for the associations between baseline FIB4 score levels and all-cause mortality.

|  |  | FIB4 low |  |  | FIB4 intermediate |  |  |  | FIB4 high |  |  |
| --- | --- | --- | --- | --- | --- | --- | --- | --- | --- | --- | --- |
|  | Subgroups | Event / total | HR (95%CI) |  | Event / total | HR (95%CI) | p |  | Event / total | HR (95%CI) | p |
| **Females** | Overall | 3739 / 41343 | 1 |  | 1180 / 11002 | 1.53 (1.40, 1.66) | <0.01 |  | 173 / 748 | 2.33 (1.91, 2.84) | <0.01 |
| Age | < 65 yrs | 1998 / 31130 | 1 |  | 972 / 10070 | 1.52 (1.41, 1.64) | <0.01 |  | 79 / 434 | 2.76 (2.2, 3.46) | <0.01 |
|  | >= 65 yrs | 1741 / 10213 | 1 |  | 208 / 932 | 1.36 (1.18, 1.57) | <0.01 |  | 94 / 314 | 1.95 (1.58, 2.4) | <0.01 |
| Townsend deprivation index | Fifth 1 (least deprived) | 573 / 7441 | 1 |  | 200 / 2031 | 1.73 (1.46, 2.04) | <0.01 |  | 22 / 119 | 2.29 (1.49, 3.52) | <0.01 |
|  | Fifth 2 | 636 / 7825 | 1 |  | 191 / 2052 | 1.41 (1.19, 1.66) | <0.01 |  | 34 / 142 | 2.73 (1.92, 3.87) | <0.01 |
|  | Fifth 3 | 680 / 8261 | 1 |  | 219 / 2131 | 1.60 (1.37, 1.88) | <0.01 |  | 30 / 140 | 2.39 (1.65, 3.45) | <0.01 |
|  | Fifth 4 | 791 / 8670 | 1 |  | 251 / 2342 | 1.40 (1.21, 1.62) | <0.01 |  | 46 / 166 | 2.61 (1.93, 3.53) | <0.01 |
|  | Fifth 5 (most deprived) | 1057 / 9097 | 1 |  | 319 / 2431 | 1.41 (1.24, 1.60) | <0.01 |  | 41 / 179 | 1.83 (1.33, 2.50) | <0.01 |
| Education | Below secondary | 1428 / 10445 | 1 |  | 448 / 3150 | 1.41 (1.26, 1.58) | <0.01 |  | 73 / 279 | 1.99 (1.57, 2.52) | <0.01 |
|  | Lower secondary | 675 / 8216 | 1 |  | 218 / 2121 | 1.51 (1.29, 1.77) | <0.01 |  | 28 / 136 | 2.20 (1.50, 3.21) | <0.01 |
|  | Higher secondary | 165 / 2173 | 1 |  | 55 / 528 | 1.75 (1.27, 2.41) | <0.01 |  | 5 / 29 | 2.07 (0.82, 5.23) | 0.12 |
|  | Vocational | 816 / 11264 | 1 |  | 275 / 2994 | 1.57 (1.36, 1.81) | <0.01 |  | 42 / 179 | 2.83 (2.06, 3.87) | <0.01 |
|  | Higher education | 653 / 9214 | 1 |  | 181 / 2200 | 1.41 (1.19, 1.67) | <0.01 |  | 25 / 125 | 2.25 (1.50, 3.39) | <0.01 |
| Smoking | Never | 1764 / 24266 | 1 |  | 554 / 6346 | 1.51 (1.37, 1.67) | <0.01 |  | 87 / 420 | 2.39 (1.93, 2.97) | <0.01 |
|  | Previous | 1369 / 13106 | 1 |  | 451 / 3758 | 1.44 (1.29, 1.61) | <0.01 |  | 63 / 269 | 2.27 (1.76, 2.92) | <0.01 |
|  | Current | 574 / 3738 | 1 |  | 159 / 822 | 1.48 (1.24, 1.78) | <0.01 |  | 22 / 54 | 2.03 (1.31, 3.14) | <0.01 |
| Physical activity | Low | 737 / 7517 | 1 |  | 222 / 1717 | 1.60 (1.37, 1.87) | <0.01 |  | 30 / 116 | 2.08 (1.43, 3.02) | <0.01 |
|  | Moderate | 935 / 11976 | 1 |  | 295 / 3198 | 1.49 (1.30, 1.70) | <0.01 |  | 38 / 191 | 2.12 (1.53, 2.94) | <0.01 |
|  | High | 649 / 8448 | 1 |  | 204 / 2378 | 1.39 (1.18, 1.63) | <0.01 |  | 35 / 174 | 2.34 (1.66, 3.30) | <0.01 |
| BMI | < 25.0 | 34 / 283 | 1 |  | 13 / 103 | 1.29 (0.62, 2.67) | 0.49 |  | 13 / 26 | 6.37 (2.55, 15.91) | <0.01 |
|  | 25.0-29.9 | 890 / 9351 | 1 |  | 269 / 2631 | 1.39 (1.21, 1.61) | <0.01 |  | 41 / 173 | 2.43 (1.77, 3.33) | <0.01 |
|  | >= 30 | 2815 / 31709 | 1 |  | 898 / 8268 | 1.51 (1.40, 1.63) | <0.01 |  | 119 / 549 | 2.07 (1.72, 2.49) | <0.01 |
| Multimorbidity | 0 LTC | 1194 / 18358 | 1 |  | 337 / 4695 | 1.48 (1.26, 1.73) | <0.01 |  | 47 / 289 | 1.97 (1.32, 2.95) | <0.01 |
|  | 1 LTC | 1112 / 12799 | 1 |  | 353 / 3431 | 1.33 (1.13, 1.57) | <0.01 |  | 46 / 223 | 2.04 (1.39, 3.01) | <0.01 |
|  | >= 2 LTCs | 1433 / 10186 | 1 |  | 490 / 2876 | 1.66 (1.45, 1.90) | <0.01 |  | 80 / 236 | 2.68 (2.01, 3.58) | <0.01 |
| Diabetes | No diabetes | 1918 / 27215 | 1 |  | 651 / 7335 | 1.41 (1.29, 1.55) | <0.01 |  | 93 / 434 | 2.37 (1.92, 2.93) | <0.01 |
|  | Diabetes | 1821 / 14128 | 1 |  | 529 / 3667 | 1.31 (1.19, 1.45) | <0.01 |  | 80 / 314 | 1.67 (1.33, 2.09) | <0.01 |
|  |  |  |  |  |  |  |  |  |  |  |  |
| **Males** | Overall | 5778 / 48692 | 1 |  | 3067 / 23667 | 1.42 (1.35, 1.50) | <0.01 |  | 534 / 2018 | 1.83 (1.65, 2.04) | <0.01 |
| Age | < 65 yrs | 2687 / 36445 | 1 |  | 2343 / 20863 | 1.49 (1.40, 1.57) | <0.01 |  | 218 / 979 | 2.92 (2.54, 3.35) | <0.01 |
|  | >= 65 yrs | 3091 / 12247 | 1 |  | 724 / 2804 | 1.07 (0.99, 1.16) | 0.10 |  | 316 / 1039 | 1.22 (1.09, 1.37) | <0.01 |
| Townsend deprivation index | Fifth 1 (least deprived) | 1031 / 10257 | 1 |  | 562 / 5292 | 1.28 (1.15, 1.42) | <0.01 |  | 98 / 422 | 1.69 (1.37, 2.09) | <0.01 |
|  | Fifth 2 | 1031 / 9983 | 1 |  | 596 / 5046 | 1.37 (1.24, 1.52) | <0.01 |  | 108 / 443 | 1.66 (1.35, 2.03) | <0.01 |
|  | Fifth 3 | 1079 / 9753 | 1 |  | 609 / 4746 | 1.44 (1.30, 1.59) | <0.01 |  | 108 / 388 | 2.08 (1.70, 2.54) | <0.01 |
|  | Fifth 4 | 1204 / 9402 | 1 |  | 605 / 4474 | 1.29 (1.16, 1.43) | <0.01 |  | 100 / 384 | 1.53 (1.25, 1.89) | <0.01 |
|  | Fifth 5 (most deprived) | 1428 / 9231 | 1 |  | 691 / 4080 | 1.33 (1.21, 1.46) | <0.01 |  | 119 / 376 | 1.52 (1.26, 1.84) | <0.01 |
| Education | Below secondary | 2106 / 10208 | 1 |  | 1047 / 5217 | 1.23 (1.14, 1.33) | <0.01 |  | 200 / 648 | 1.36 (1.17, 1.57) | <0.01 |
|  | Lower secondary | 721 / 6966 | 1 |  | 376 / 3078 | 1.39 (1.23, 1.59) | <0.01 |  | 52 / 196 | 2.01 (1.50, 2.69) | <0.01 |
|  | Higher secondary | 221 / 2382 | 1 |  | 121 / 1104 | 1.58 (1.25, 2.00) | <0.01 |  | 14 / 74 | 1.03 (0.59, 1.80) | 0.92 |
|  | Vocational | 1585 / 14947 | 1 |  | 876 / 7220 | 1.37 (1.26, 1.49) | <0.01 |  | 140 / 583 | 1.80 (1.51, 2.14) | <0.01 |
|  | Higher education | 1134 / 14151 | 1 |  | 645 / 7031 | 1.37 (1.24, 1.51) | <0.01 |  | 126 / 515 | 2.19 (1.81, 2.64) | <0.01 |
| Smoking | Never | 1891 / 23837 | 1 |  | 1064 / 11485 | 1.36 (1.26, 1.47) | <0.01 |  | 198 / 907 | 1.92 (1.65, 2.22) | <0.01 |
|  | Previous | 2884 / 18840 | 1 |  | 1562 / 9983 | 1.29 (1.21, 1.38) | <0.01 |  | 282 / 927 | 1.53 (1.35, 1.73) | <0.01 |
|  | Current | 944 / 5725 | 1 |  | 413 / 2057 | 1.40 (1.24, 1.58) | <0.01 |  | 50 / 163 | 1.75 (1.31, 2.34) | <0.01 |
| Physical activity | Low | 1196 / 9668 | 1 |  | 628 / 4151 | 1.47 (1.33, 1.63) | <0.01 |  | 119 / 345 | 2.10 (1.73, 2.55) | <0.01 |
|  | Moderate | 1642 / 15196 | 1 |  | 909 / 7330 | 1.36 (1.25, 1.47) | <0.01 |  | 137 / 577 | 1.66 (1.39, 1.98) | <0.01 |
|  | High | 1408 / 13595 | 1 |  | 770 / 7318 | 1.28 (1.17, 1.40) | <0.01 |  | 134 / 623 | 1.50 (1.25, 1.79) | <0.01 |
| BMI | < 25.0 | 152 / 1365 | 1 |  | 67 / 585 | 1.30 (0.96, 1.76) | 0.10 |  | 28 / 73 | 3.02 (1.97, 4.65) | <0.01 |
|  | 25.0-29.9 | 2478 / 24316 | 1 |  | 1289 / 11698 | 1.36 (1.26, 1.45) | <0.01 |  | 227 / 920 | 1.75 (1.52, 2.01) | <0.01 |
|  | >= 30 | 3148 / 23011 | 1 |  | 1711 / 11384 | 1.32 (1.24, 1.41) | <0.01 |  | 279 / 1025 | 1.52 (1.34, 1.72) | <0.01 |
| Multimorbidity | 0 LTC | 2120 / 26707 | 1 |  | 1099 / 12255 | 1.40 (1.28, 1.53) | <0.01 |  | 133 / 812 | 1.53 (1.25, 1.89) | <0.01 |
|  | 1 LTC | 1620 / 13440 | 1 |  | 870 / 6745 | 1.38 (1.25, 1.52) | <0.01 |  | 164 / 616 | 1.91 (1.58, 2.31) | <0.01 |
|  | >= 2 LTCs | 2038 / 8545 | 1 |  | 1098 / 4667 | 1.32 (1.20, 1.44) | <0.01 |  | 237 / 590 | 1.74 (1.48, 2.05) | <0.01 |
| Diabetes | No diabetes | 3008 / 34481 |  |  | 1766 / 16984 | 1.21 (1.14, 1.29) | <0.01 |  | 296 / 1285 | 1.60 (1.41, 1.81) | <0.01 |
|  | Diabetes | 2770 / 14211 | 1 |  | 1301 / 6683 | 1.18 (1.10, 1.27) | <0.01 |  | 238 / 733 | 1.40 (1.23, 1.60) | <0.01 |

The Cox model was stratified by region and age group, adjusted for sex, ethnicity, education, Townsend Deprivation Index (in fifths), physical activity, smoking status, body mass index levels and weekly alcohol consumption. The HRs were estimated with people with 0 LTC as reference in separate models for MASLD and non-MASLD cohorts. FIB-4 score levels were defined using the cutoff values 1.30 and 2.67 to categorise low, intermediate and high levels for people < 65 years old, and 2.00 and 2.67 for people >= 65 years old.

## Supplementary table 6: Associations between baseline FIB4 score levels and all-cause mortality, with and without additional adjustment for long-term conditions.

|  | FIB4  intermediate vs. low |  | FIB4  high vs. low |  |
| --- | --- | --- | --- | --- |
| Long-term condition additionally adjusted for | HR (95%CI) | % reduction in coefficient estimates | HR (95%CI) | % reduction in coefficient estimates |
| **Overall** |  |  |  |  |
| Without adjustment for long-term condition | 1.45 (1.38, 1.51) |  | 1.94 (1.77, 2.13) |  |
| Additionally adjusted for the condition |  |  |  |  |
| Congenital diseases | 1.45 (1.38, 1.51) | 0.1 | 1.94 (1.77, 2.13) | 0.3 |
| Chromosomal abnormalities | 1.45 (1.38, 1.51) | 0 | 1.94 (1.77, 2.13) | 0 |
| HIV/AIDS | 1.45 (1.38, 1.51) | 0 | 1.94 (1.77, 2.13) | 0 |
| Chronic urinary tract infection | 1.45 (1.38, 1.51) | 0 | 1.94 (1.77, 2.13) | 0.1 |
| Tuberculosis | 1.45 (1.38, 1.51) | 0 | 1.94 (1.77, 2.13) | 0.1 |
| Parkinson's disease | 1.44 (1.38, 1.51) | 1 | 1.95 (1.77, 2.14) | -0.3 |
| Dementia | 1.45 (1.38, 1.51) | 0.1 | 1.94 (1.77, 2.13) | -0.1 |
| Schizophrenia | 1.45 (1.38, 1.51) | -0.1 | 1.94 (1.77, 2.13) | 0.1 |
| Depression | 1.45 (1.39, 1.52) | -0.3 | 1.94 (1.77, 2.13) | 0 |
| Anxiety | 1.45 (1.38, 1.51) | 0 | 1.94 (1.77, 2.13) | 0 |
| Bipolar disorder | 1.45 (1.38, 1.51) | 0.1 | 1.94 (1.77, 2.13) | 0.1 |
| Substance use disorder | 1.45 (1.38, 1.51) | -0.2 | 1.94 (1.76, 2.12) | 0.5 |
| Eating disorder | 1.45 (1.38, 1.51) | 0 | 1.94 (1.77, 2.13) | 0 |
| Autism | 1.45 (1.38, 1.51) | -0.1 | 1.94 (1.77, 2.13) | 0 |
| PTSD | 1.45 (1.38, 1.51) | 0 | 1.94 (1.77, 2.13) | 0 |
| **Ischemic heart disease** | 1.4 (1.34, 1.46) | 9.5 | 1.85 (1.69, 2.03) | 7.1 |
| **Heart failure** | 1.42 (1.36, 1.49) | 4.2 | 1.87 (1.7, 2.05) | 5.8 |
| **Stroke** | 1.44 (1.38, 1.51) | 1 | 1.93 (1.76, 2.12) | 1.1 |
| TIA | 1.45 (1.38, 1.51) | 0.2 | 1.94 (1.77, 2.13) | 0 |
| COPD | 1.45 (1.38, 1.51) | 0 | 1.93 (1.76, 2.12) | 0.7 |
| Chronic respiratory disease | 1.45 (1.38, 1.51) | -0.2 | 1.94 (1.77, 2.13) | -0.1 |
| Asthma | 1.45 (1.39, 1.52) | -1.1 | 1.95 (1.78, 2.14) | -0.5 |
| **Solid organ cancers** | 1.43 (1.36, 1.49) | 4 | 1.92 (1.75, 2.11) | 1.5 |
| **Haematological cancers** | 1.44 (1.38, 1.51) | 0.9 | 1.91 (1.74, 2.1) | 2.5 |
| Addison's disease | 1.45 (1.38, 1.51) | 0 | 1.94 (1.77, 2.13) | -0.1 |
| Cystic fibrosis | 1.45 (1.38, 1.51) | 0 | 1.94 (1.77, 2.13) | 0 |
| Epilepsy | 1.45 (1.38, 1.51) | -0.2 | 1.94 (1.77, 2.13) | 0 |
| Multiple sclerosis | 1.45 (1.38, 1.51) | -0.1 | 1.94 (1.77, 2.13) | -0.1 |
| Paralysis | 1.45 (1.38, 1.51) | -0.1 | 1.95 (1.77, 2.14) | -0.3 |
| Connective tissue disease | 1.44 (1.38, 1.51) | 0.5 | 1.93 (1.76, 2.12) | 0.7 |
| Irritable bowel disease | 1.45 (1.38, 1.51) | 0.1 | 1.94 (1.77, 2.13) | 0.2 |
| **Chronic kidney disease** | 1.44 (1.37, 1.5) | 1.7 | 1.92 (1.75, 2.1) | 2 |
| **Heart valve disorder** | 1.44 (1.38, 1.5) | 1.7 | 1.91 (1.74, 2.09) | 2.7 |
| **Arrythmia** | 1.43 (1.36, 1.49) | 4 | 1.87 (1.7, 2.05) | 5.9 |
| Venous thromboembolism | 1.44 (1.38, 1.51) | 0.5 | 1.93 (1.76, 2.12) | 0.8 |
| Aneurysm | 1.45 (1.38, 1.51) | 0 | 1.94 (1.77, 2.13) | 0.2 |
| Thyroid disorder | 1.45 (1.38, 1.51) | 0.3 | 1.94 (1.77, 2.13) | 0.1 |
| Osteoarthritis | 1.44 (1.38, 1.51) | 0.5 | 1.94 (1.77, 2.13) | 0.2 |
| Osteoporosis | 1.45 (1.38, 1.51) | 0.2 | 1.95 (1.78, 2.14) | -0.6 |
| Gout | 1.44 (1.38, 1.51) | 0.5 | 1.93 (1.76, 2.12) | 0.7 |
| Chronic pancreatitis | 1.45 (1.38, 1.51) | 0 | 1.94 (1.77, 2.13) | -0.1 |
| Peptic ulcer disease | 1.45 (1.38, 1.51) | 0.1 | 1.94 (1.77, 2.13) | 0 |
| Endometriosis | 1.45 (1.38, 1.51) | 0.2 | 1.94 (1.77, 2.13) | 0.1 |
| Anaemia | 1.45 (1.38, 1.51) | 0.1 | 1.94 (1.77, 2.13) | 0.3 |
| Vision impairment | 1.44 (1.38, 1.51) | 0.7 | 1.96 (1.78, 2.15) | -1 |
| Hearing impairment | 1.45 (1.38, 1.51) | 0 | 1.94 (1.77, 2.13) | -0.1 |
| Meniere's disease | 1.45 (1.38, 1.51) | 0 | 1.94 (1.77, 2.13) | 0 |
|  |  |  |  |  |
| **Females** |  |  |  |  |
| Without adjustment forlong-term condition | 1.53 (1.40, 1.66) |  | 2.33 (1.91, 2.84) |  |
| Additionally adjusted for the condition |  |  |  |  |
| Congenital diseases | 1.53 (1.4, 1.66) | -0.2 | 2.33 (1.91, 2.84) | 0.1 |
| Chromosomal abnormalities | 1.53 (1.4, 1.66) | 0 | 2.33 (1.91, 2.84) | 0 |
| HIV/AIDS | 1.53 (1.4, 1.66) | 0 | 2.33 (1.91, 2.84) | 0 |
| Chronic urinary tract infection | 1.53 (1.4, 1.66) | 0 | 2.33 (1.91, 2.84) | 0 |
| Tuberculosis | 1.52 (1.4, 1.66) | 0.2 | 2.32 (1.9, 2.84) | 0.2 |
| Parkinson's disease | 1.52 (1.4, 1.66) | 0.4 | 2.31 (1.89, 2.82) | 1 |
| Dementia | 1.53 (1.4, 1.66) | 0 | 2.33 (1.91, 2.84) | 0.1 |
| Schizophrenia | 1.53 (1.4, 1.66) | 0 | 2.33 (1.91, 2.84) | 0 |
| Depression | 1.53 (1.4, 1.67) | -0.5 | 2.33 (1.91, 2.85) | -0.2 |
| Anxiety | 1.53 (1.4, 1.66) | 0 | 2.33 (1.91, 2.84) | -0.1 |
| Bipolar disorder | 1.53 (1.4, 1.66) | 0 | 2.32 (1.9, 2.83) | 0.4 |
| Substance use disorder | 1.53 (1.4, 1.66) | 0 | 2.32 (1.9, 2.83) | 0.5 |
| Eating disorder | 1.52 (1.4, 1.66) | 0.1 | 2.33 (1.91, 2.84) | -0.1 |
| Autism | 1.53 (1.4, 1.66) | -0.1 | 2.33 (1.91, 2.84) | 0 |
| PTSD | 1.53 (1.4, 1.66) | 0 | 2.33 (1.91, 2.84) | 0 |
| Ischemic heart disease | 1.49 (1.37, 1.63) | 4.9 | 2.29 (1.87, 2.79) | 2 |
| Heart failure | 1.51 (1.39, 1.65) | 2.4 | 2.32 (1.9, 2.83) | 0.6 |
| Stroke | 1.52 (1.39, 1.66) | 1.1 | 2.32 (1.9, 2.84) | 0.2 |
| TIA | 1.53 (1.4, 1.66) | 0 | 2.33 (1.91, 2.84) | -0.1 |
| COPD | 1.52 (1.4, 1.66) | 0.6 | 2.34 (1.92, 2.86) | -0.8 |
| Chronic respiratory disease | 1.53 (1.4, 1.67) | -0.2 | 2.32 (1.9, 2.83) | 0.6 |
| Asthma | 1.54 (1.41, 1.67) | -1.4 | 2.34 (1.91, 2.85) | -0.5 |
| Solid organ cancers | 1.48 (1.36, 1.62) | 6.7 | 2.24 (1.84, 2.74) | 4.5 |
| Haematological cancers | 1.52 (1.4, 1.66) | 0.3 | 2.32 (1.9, 2.84) | 0.1 |
| Addison's disease | 1.53 (1.4, 1.67) | -0.2 | 2.33 (1.91, 2.84) | -0.2 |
| Cystic fibrosis | 1.53 (1.4, 1.66) | 0 | 2.33 (1.91, 2.84) | 0 |
| Epilepsy | 1.53 (1.4, 1.67) | -0.2 | 2.33 (1.91, 2.85) | -0.4 |
| Multiple sclerosis | 1.53 (1.4, 1.66) | 0 | 2.33 (1.91, 2.84) | 0 |
| Paralysis | 1.53 (1.4, 1.66) | -0.1 | 2.33 (1.91, 2.84) | -0.1 |
| Connective tissue disease | 1.52 (1.39, 1.66) | 1.1 | 2.32 (1.9, 2.83) | 0.6 |
| Irritable bowel disease | 1.53 (1.4, 1.66) | 0 | 2.33 (1.91, 2.84) | 0 |
| Chronic kidney disease | 1.52 (1.39, 1.66) | 1.1 | 2.33 (1.91, 2.84) | -0.1 |
| Heart valve disorder | 1.52 (1.39, 1.65) | 1.4 | 2.33 (1.91, 2.84) | 0 |
| Arrythmia | 1.52 (1.39, 1.65) | 1.5 | 2.31 (1.89, 2.82) | 1.1 |
| Venous thromboembolism | 1.52 (1.4, 1.66) | 0.2 | 2.32 (1.9, 2.83) | 0.3 |
| Aneurysm | 1.53 (1.4, 1.66) | 0 | 2.33 (1.91, 2.84) | 0.1 |
| Thyroid disorder | 1.52 (1.4, 1.66) | 0.6 | 2.32 (1.9, 2.83) | 0.3 |
| Osteoarthritis | 1.52 (1.4, 1.66) | 0.3 | 2.31 (1.9, 2.83) | 0.7 |
| Osteoporosis | 1.52 (1.4, 1.66) | 0.5 | 2.34 (1.91, 2.85) | -0.5 |
| Gout | 1.52 (1.4, 1.66) | 0.6 | 2.33 (1.91, 2.85) | -0.4 |
| Chronic pancreatitis | 1.53 (1.4, 1.67) | -0.2 | 2.33 (1.91, 2.84) | -0.2 |
| Peptic ulcer disease | 1.53 (1.4, 1.66) | 0 | 2.33 (1.91, 2.84) | 0 |
| Endometriosis | 1.52 (1.39, 1.66) | 0.8 | 2.32 (1.9, 2.83) | 0.6 |
| Anaemia | 1.53 (1.4, 1.67) | -0.2 | 2.32 (1.9, 2.84) | 0.2 |
| Vision impairment | 1.52 (1.4, 1.66) | 0.6 | 2.34 (1.92, 2.86) | -0.7 |
| Hearing impairment | 1.53 (1.4, 1.66) | 0.1 | 2.33 (1.91, 2.84) | 0 |
| Meniere's disease | 1.53 (1.4, 1.66) | 0 | 2.33 (1.91, 2.84) | 0.1 |
|  |  |  |  |  |
| **Males** |  |  |  |  |
| Without adjustment forlong-term condition | 1.42 (1.35, 1.50) |  | 1.83 (1.65, 2.04) |  |
| Additionally adjusted for the condition |  |  |  |  |
| Congenital diseases | 1.42 (1.35, 1.49) | 0.3 | 1.83 (1.65, 2.03) | 0.4 |
| Chromosomal abnormalities | 1.42 (1.35, 1.49) | 0.1 | 1.83 (1.65, 2.04) | 0 |
| HIV/AIDS | 1.42 (1.35, 1.5) | 0 | 1.83 (1.65, 2.04) | 0 |
| Chronic urinary tract infection | 1.42 (1.35, 1.5) | 0 | 1.83 (1.65, 2.03) | 0.3 |
| Tuberculosis | 1.42 (1.35, 1.5) | -0.1 | 1.83 (1.65, 2.04) | 0.1 |
| Parkinson's disease | 1.41 (1.34, 1.49) | 1.2 | 1.84 (1.66, 2.04) | -0.5 |
| Dementia | 1.42 (1.35, 1.49) | 0.2 | 1.84 (1.65, 2.04) | -0.2 |
| Schizophrenia | 1.42 (1.35, 1.5) | -0.1 | 1.83 (1.65, 2.04) | 0.2 |
| Depression | 1.42 (1.35, 1.5) | -0.2 | 1.83 (1.65, 2.04) | 0 |
| Anxiety | 1.42 (1.35, 1.5) | 0 | 1.84 (1.65, 2.04) | -0.1 |
| Bipolar disorder | 1.42 (1.35, 1.49) | 0.1 | 1.83 (1.65, 2.04) | 0.1 |
| Substance use disorder | 1.42 (1.35, 1.5) | -0.2 | 1.83 (1.64, 2.03) | 0.6 |
| Eating disorder | 1.42 (1.35, 1.5) | 0 | 1.83 (1.65, 2.04) | 0 |
| Autism | 1.42 (1.35, 1.5) | -0.1 | 1.83 (1.65, 2.04) | 0 |
| PTSD | 1.42 (1.35, 1.5) | 0 | 1.83 (1.65, 2.04) | 0 |
| Ischemic heart disease | 1.36 (1.29, 1.44) | 11.3 | 1.74 (1.57, 1.93) | 8.5 |
| Heart failure | 1.39 (1.32, 1.47) | 5.2 | 1.76 (1.58, 1.95) | 7.2 |
| Stroke | 1.41 (1.34, 1.49) | 0.9 | 1.82 (1.64, 2.02) | 1.3 |
| TIA | 1.42 (1.35, 1.49) | 0.3 | 1.83 (1.65, 2.04) | 0.1 |
| COPD | 1.42 (1.35, 1.5) | -0.2 | 1.82 (1.64, 2.03) | 0.9 |
| Chronic respiratory disease | 1.42 (1.35, 1.5) | -0.1 | 1.84 (1.65, 2.04) | -0.2 |
| Asthma | 1.42 (1.35, 1.5) | -0.9 | 1.84 (1.66, 2.04) | -0.4 |
| Solid organ cancers | 1.4 (1.33, 1.48) | 2.9 | 1.83 (1.65, 2.03) | 0.4 |
| Haematological cancers | 1.41 (1.34, 1.49) | 1.1 | 1.8 (1.62, 2) | 3.3 |
| Addison's disease | 1.42 (1.35, 1.5) | 0 | 1.83 (1.65, 2.04) | 0.1 |
| Cystic fibrosis | 1.42 (1.35, 1.5) | 0 | 1.83 (1.65, 2.04) | 0 |
| Epilepsy | 1.42 (1.35, 1.5) | -0.2 | 1.83 (1.65, 2.03) | 0.2 |
| Multiple sclerosis | 1.42 (1.35, 1.5) | -0.3 | 1.84 (1.65, 2.04) | -0.2 |
| Paralysis | 1.42 (1.35, 1.5) | -0.1 | 1.84 (1.65, 2.04) | -0.3 |
| Connective tissue disease | 1.42 (1.35, 1.49) | 0.2 | 1.83 (1.65, 2.03) | 0.6 |
| Irritable bowel disease | 1.42 (1.35, 1.49) | 0.1 | 1.83 (1.65, 2.04) | 0.2 |
| Chronic kidney disease | 1.41 (1.34, 1.48) | 2.1 | 1.81 (1.62, 2.01) | 2.6 |
| Heart valve disorder | 1.41 (1.34, 1.49) | 1.8 | 1.79 (1.61, 1.99) | 3.8 |
| Arrythmia | 1.39 (1.32, 1.47) | 5.2 | 1.75 (1.57, 1.94) | 8.2 |
| Venous thromboembolism | 1.42 (1.34, 1.49) | 0.6 | 1.83 (1.64, 2.03) | 0.8 |
| Aneurysm | 1.42 (1.35, 1.49) | 0.1 | 1.83 (1.65, 2.04) | 0.1 |
| Thyroid disorder | 1.42 (1.35, 1.49) | 0.2 | 1.83 (1.65, 2.04) | 0 |
| Osteoarthritis | 1.42 (1.34, 1.49) | 0.5 | 1.83 (1.65, 2.04) | 0 |
| Osteoporosis | 1.42 (1.35, 1.49) | 0.1 | 1.84 (1.66, 2.04) | -0.6 |
| Gout | 1.42 (1.34, 1.49) | 0.5 | 1.82 (1.64, 2.03) | 0.9 |
| Chronic pancreatitis | 1.42 (1.35, 1.49) | 0.1 | 1.84 (1.65, 2.04) | -0.1 |
| Peptic ulcer disease | 1.42 (1.35, 1.49) | 0.1 | 1.84 (1.65, 2.04) | -0.1 |
| Endometriosis | 1.42 (1.35, 1.5) | 0 | 1.83 (1.65, 2.04) | 0 |
| Anaemia | 1.42 (1.34, 1.49) | 0.3 | 1.83 (1.65, 2.03) | 0.4 |
| Vision impairment | 1.42 (1.34, 1.49) | 0.8 | 1.85 (1.66, 2.05) | -1.2 |
| Hearing impairment | 1.42 (1.35, 1.5) | 0 | 1.84 (1.65, 2.04) | -0.1 |
| Meniere's disease | 1.42 (1.35, 1.5) | 0 | 1.84 (1.65, 2.04) | -0.1 |
